# Serum metabolites and risk of myocardial infarction and ischemic stroke: a targeted metabolomic approach in two German prospective cohorts

**DOI:** 10.1007/s10654-017-0333-0

**Published:** 2017-11-27

**Authors:** Anna Floegel, Tilman Kühn, Disorn Sookthai, Theron Johnson, Cornelia Prehn, Ulrike Rolle-Kampczyk, Wolfgang Otto, Cornelia Weikert, Thomas Illig, Martin von Bergen, Jerzy Adamski, Heiner Boeing, Rudolf Kaaks, Tobias Pischon

**Affiliations:** 1 Department of Epidemiology, German Institute of Human Nutrition Potsdam-Rehbruecke, Nuthetal, Germany; 2 Leibniz Institute for Prevention Research and Epidemiology – BIPS, Achterstraße 30, 28359 Bremen, Germany; 3 Division of Cancer Epidemiology, German Cancer Research Center (DKFZ), Heidelberg, Germany; 4 Institute of Experimental Genetics, Helmholtz Zentrum München, German Research Center for Environmental Health, Neuherberg, Germany; 5 Department of Molecular Systems Biology, Helmholtz Centre for Environmental Research (UFZ), Leipzig, Germany; 6 Department of Food Safety, Federal Institute for Risk Assessment, Berlin, Germany; 7 Institute for Social Medicine, Epidemiology and Health Economics, Charité University Medical Center, Berlin, Germany; 8 Hannover Unified Biobank, Hannover Medical School, Hannover, Germany; 9 Institute of Human Genetics, Hannover Medical School, Hannover, Germany; 10 University of Aalborg, Fredrik Bajers Vej 7H, 9220 Aalborg East, Denmark; 11 Molecular Epidemiology Group, Max Delbrück Center for Molecular Medicine (MDC), Berlin, Germany; 12 Charité – Universitätsmedizin Berlin, Berlin, Germany; 13 German Center for Cardiovascular Research (DZHK), Partner Site Berlin, Berlin, Germany

**Keywords:** Metabolomics, Myocardial infarction, Stroke, Biomarker, Prospective cohort study

## Abstract

Metabolomic approaches in prospective cohorts may offer a unique snapshot into early metabolic perturbations that are associated with a higher risk of cardiovascular diseases (CVD) in healthy people. We investigated the association of 105 serum metabolites, including acylcarnitines, amino acids, phospholipids and hexose, with risk of myocardial infarction (MI) and ischemic stroke in the European Prospective Investigation into Cancer and Nutrition (EPIC)-Potsdam (27,548 adults) and Heidelberg (25,540 adults) cohorts. Using case-cohort designs, we measured metabolites among individuals who were free of CVD and diabetes at blood draw but developed MI (n = 204 and n = 228) or stroke (n = 147 and n = 121) during follow-up (mean, 7.8 and 7.3 years) and among randomly drawn subcohorts (n = 2214 and n = 770). We used Cox regression analysis and combined results using meta-analysis. Independent of classical CVD risk factors, ten metabolites were associated with risk of MI in both cohorts, including sphingomyelins, diacyl-phosphatidylcholines and acyl-alkyl-phosphatidylcholines with pooled relative risks in the range of 1.21–1.40 per one standard deviation increase in metabolite concentrations. The metabolites showed positive correlations with total- and LDL-cholesterol (r ranged from 0.13 to 0.57). When additionally adjusting for total-, LDL- and HDL-cholesterol, triglycerides and C-reactive protein, acyl-alkyl-phosphatidylcholine C36:3 and diacyl-phosphatidylcholines C38:3 and C40:4 remained associated with risk of MI. When added to classical CVD risk models these metabolites further improved CVD prediction (c-statistics increased from 0.8365 to 0.8384 in EPIC-Potsdam and from 0.8344 to 0.8378 in EPIC-Heidelberg). None of the metabolites was consistently associated with stroke risk. Alterations in sphingomyelin and phosphatidylcholine metabolism, and particularly metabolites of the arachidonic acid pathway are independently associated with risk of MI in healthy adults.

**Electronic supplementary material:**

The online version of this article (10.1007/s10654-017-0333-0) contains supplementary material, which is available to authorized users.

## Introduction

A better understanding of the pathophysiological mechanisms preceding the onset of cardiovascular disease (CVD) events is crucial for development of preventive strategies and treatment options. Thereby, particularly early metabolic alterations that already occur in healthy individuals may be identified as targets for measures to delay or prevent disease onset. Metabolomic approaches that simultaneously measure substrates, intermediate- and end-products of metabolism offer a unique snapshot of metabolic perturbations that may be involved in the development of CVD [1, 2]. In this context, circulating metabolite concentrations may be altered years before the onset of CVD events.

Previous prospective metabolomic studies have identified a number of metabolites linked to risk of CVD recurrence or death in patient cohorts, as well as CVD risk in high-risk populations [3–5]. They reported an altered metabolism of acylcarnitines, ketone-related metabolites, fatty acids, choline and its phospholipids in CVD patients and high-risk individuals. One recent prospective study [6] reported phenylalanine and fatty acids, and another study [7] found other lipid species to be linked to CVD risk in population-based cohorts. Ganna et al. [8] recently found four lipid metabolites that could be useful to predict coronary heart disease. These previous studies, however, also had some limitations, e.g. they investigated few metabolite classes or a composite CVD endpoint. Thus, there is an urgent need for large metabolomic studies covering a wide range of metabolites that are conducted in healthy adult cohorts, which are followed over long for incidence of a first CVD event. In addition, it is of great importance to address different CVD endpoints separately, so as to better understand their individual pathophysiological mechanisms.

The present study aimed to identify metabolites, which are linked to higher incidence of myocardial infarction (MI) and ischemic stroke in initially healthy adults. Therefore, we conducted targeted metabolomic measurements, including in total 105 metabolites among amino acids, acylcarnitines, phosphatidylcholines, sphingomyelins and hexose, in serum samples from two large prospective cohorts comprising middle-aged adults from Germany that were healthy at the time of the blood sample collection and followed for development of a prime CVD event. To better understand the biological mechanisms, in addition, we studied associations between metabolites and established biomarkers of CVD risk. To evaluate their usefulness for clinical practice we also calculated measures of risk prediction.

## Methods

### Study population

The present study is based on data from the European Prospective Investigation into Cancer and Nutrition (EPIC) Germany study, a prospective cohort study which includes 27,548 adults in Potsdam and 25,540 adults in Heidelberg aged mainly between 35 and 65 years at time of recruitment, when also the blood sample was collected [9]. These people are prospectively followed for incidence of chronic diseases including CVD. More general details about the cohorts are provided in the Supplementary Methods.

**Fig. 1:**
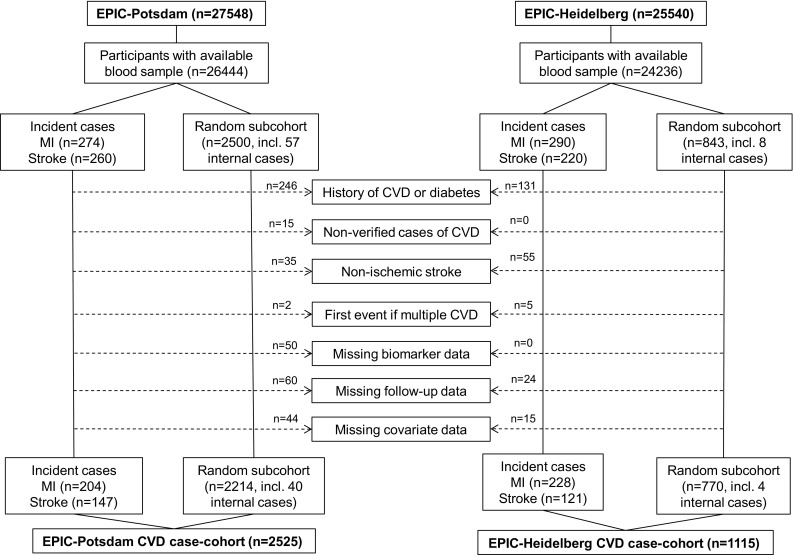
Flow diagram of participants’ selection from the two original cohorts

We constructed two case-cohort studies, one in EPIC-Potsdam and another in EPIC-Heidelberg, including all incident cases of MI (n = 274 and n = 290; respectively) and stroke (n = 260 and n = 220; respectively) that occurred in the full cohorts until December 2006, after mean follow-up time of 7.8 years (Potsdam) and 7.3 years (Heidelberg), and two randomly drawn subcohorts (Potsdam n = 2500, Heidelberg n = 843) from all participants who had provided blood samples in the full cohorts. The selection of the subcohorts has been described in detail previously [10, 11]. For the present analysis, the following additional exclusion criteria were applied (Fig. 1): history of CVD or diabetes mellitus at the time of blood donation (to ensure that initially healthy adults were included), non-verified incident CVD, non-ischemic incident stroke, missing biomarker data or missing covariates. Thus, the final study sample in EPIC-Potsdam consisted of 204 incident cases of MI and 147 incident cases of stroke, and a random subcohort of n = 2214. From EPIC-Heidelberg, 228 incident cases of MI and 121 incident cases of stroke were considered, in addition to a random subcohort of n = 770.

### Ascertainment of incident MI and stroke during follow-up

Potential incident cases of MI and stroke were identified based on self-reports of a new diagnosis of disease, disease relevant medication or change in diet due to disease, which were collected every 2–3 years after baseline in follow-up questionnaires [12]. In addition, information from death certificates or linkage to a hospital information system of the major hospital in the area was considered. Follow-up rounds resulted in response rates of about 95% of participants [12, 13]. Self-reported cases were further verified by actively contacting the treating physician or hospital who filled in a standard inquiry form that included information on the exact type and date of diagnosis, the method of confirmation of the diagnosis and treatment information. We used the international classification of diseases (ICD)-10 system to classify incident cases: I21 for MI, I60 and I61 for haemorrhagic stroke, I63 for ischemic stroke and I64 for undetermined stroke.

### Metabolomic measurements

Metabolite concentrations were determined in baseline serum samples of the EPIC-Potsdam and EPIC-Heidelberg case-cohort studies using two commercial kits (BIOCRATES Life Sciences AG, Innsbruck, Austria). Blood samples were stored in liquid nitrogen until analysis. Sample preparation was done according to standardized protocols and the metabolomic methods have been described in detail elsewhere [14, 15]. Details are provided in the Supplementary Methods.

The p150 metabolomic kit used in EPIC-Potsdam initially contained 163 metabolites, of them 14 amino acids, 41 acylcarnitines, 1 hexose, 92 glycerophospholipids (phosphatidylcholines and lyso-phosphatidylcholines) and 15 sphingomyelins; a detailed list has been published earlier by the authors [11]. A new version of the kit including 188 metabolites (similar metabolites but additionally including biogenic amines) was used for measurement of EPIC-Heidelberg samples. For the present analysis we considered only metabolites that were measured in both studies (n = 161). Data pre-processing was done as previously described [16]. In brief, we excluded metabolite species with more than 25% values below limit of detection, with more than 25% missing values, and with batch variation of more than 25%. All metabolite values were log2 transformed and normalized by metabolite wide batch standardization [17]. Robust principal component analysis was used to identify multivariable outliers, which were excluded [18]. This led to inclusion of a total of 105 metabolites, of them 13 amino acids, 2 acylcarnitines, 1 hexose, 77 glycerophospholipids and 12 sphingomyelins into the present analysis.

### Statistical analysis

Baseline characteristics of both study populations were calculated as age- and sex-adjusted mean and standard error (continuous variables) or percentages (categorical variables). Serum metabolite concentrations were standardized (mean of 0 and SD of 1), to make them directly comparable, and log2 transformed to better approximate the normal distribution; and serum metabolite concentrations according to case status were calculated as geometric mean and 95% confidence interval (CI).

In both case-cohort studies, we used Cox proportional hazard regression with weighting as suggested by Prentice [19] and robust sandwich covariance estimates to calculate hazard rate ratios and 95% CI, considering serum metabolite concentrations as the exposure variable and diagnosis of MI or stroke as the outcome, with age of each participant as the underlying time-scale from entry (baseline) to exit time (diagnosis of MI or stroke or censoring or death) in the study. We calculated a multivariable adjusted model considering the following covariates: age; sex; education (no degree/vocational training; trade/technical school; university degree); smoking (never, former, current ≤ 20 cigarettes/day, current heavy > 20 cigarettes/day); alcohol intake (non-consumers, women: > 0–6 g/day, 6–12 g/day, > 12 g/day; men: > 0–12 g/day, 12–24 g/day, > 24 g/day); physical activity (Potsdam: cycling and sports in h/week; Heidelberg: Cambridge physical activity index); fasting status (y/n); waist circumference (cm); BMI (kg/m^2^); and prevalent hypertension (y/n). *P* values were corrected for multiple testing by controlling the false discovery rate [20]. We ran separate analyses for each endpoint and each study population.

As the selection of metabolites very much depends on the *p* value treshold, we used a different approach to identify metabolites that does not so much rely on the method used for multiple testing correction. We applied a meta-analytical approach in the beginning and only considered those metabolites that were associated with risk of MI or stroke in both study populations. So reproducibility of associations was our key selection factor for identification of metabolites. For the meta-analysis, random effects model were calculated and heterogeneity was assessed by measures of I^2^ [21]. For the selected metabolites, we calculated further Cox-regression models additionally adjusting for intake of lipid lowering medication (statin and fibrate intake) and established CVD biomarkers, including total cholesterol, HDL-cholesterol, LDL-Cholesterol, triglycerides and high-sensitivity C-reactive protein (hs-CRP). As LDL-Cholesterol was not measured it was estimated from the other lipids using the Friedewald formula [22]. In addition, we calculated Spearman partial correlation coefficients between metabolites and established CVD biomarkers, adjusted for age and sex. In a sensitivity analysis, we calculated hazard rates across different follow-up periods (≤ 3 years, 3–6 years, > 6 years) for selected metabolites. We then tested heterogeneity according to Hardy and Thompson [21].

To investigate whether the metabolites are useful to predict myocardial infarction, we calculated measures of discrimination (c-statistic [23]) and calibration (Hosmer–Lemeshow test [24]) for selected metabolites and established CVD biomarkers with logistic regression models. We drew receiver operating characteristic (ROC) curves [23] for comparison of different models when adding selected metabolites to established risk factors and biomarkers.

The meta-analysis was conducted in R (version 3.2.1) using the Metagen-package. All other analyses were conducted with SAS enterprise guide (version 6.1, SAS Institute Inc., Cary, NC,USA).

## Results

Baseline characteristics of the study participants are presented in Table 1. Mean age of participants from both subcohorts was about 49 years. In general, participants who developed CVD were older, less likely to be female and lifestyle factors and biomarkers were more unfavourable compared to the subcohorts.

**Table 1:** Baseline characteristics^a^ of the study cohorts (1994–1998)

	EPIC-Potsdam			EPIC-Heidelberg		
	Random subcohort (n = 2214)	Incident MI (n = 204)	Incident stroke (n = 147)	Random subcohort (n = 770)	Incident MI (n = 228)	Incident stroke (n = 121)
Age (years)^b^	49.2 (8.9)	55.1 (7.2)	55.1 (8.0)	49.8 (8.0)	54.8 (6.3)	55.0 (7.3)
Women (%)^b^	63.1	28.4	49.7	56.9	19.3	38.0
BMI (kg/m^2^)	26.0 (0.1)	26.6 (0.3)	26.4 (0.3)	25.7 (0.1)	27.0 (0.3)	26.1 (0.4)
Waist circumference, men (cm)^c^	93.7 (0.3)	96.2 (0.8)	94.9 (1.1)	95.3 (0.5)	98.5 (0.7)	94.8 (1.1)
Waist circumference, women (cm)^c^	80.4 (0.3)	81.7 (1.4)	80.9 (1.3)	79.8 (0.5)	82.4 (1.7)	82.4 (1.6)
History of hypertension (%)	47.2	57.3	62.7	28.2	40.5	38.5
Education						
No degree/vocational training (%)	36.9	38.6	40.7	26.7	32.9	34.7
Trade/technical school (%)	24.0	25.1	30.1	41.4	40.9	38.1
University degree (%)	39.1	36.3	29.2	31.9	26.2	27.2
Smoking status						
Never (%)	47.6	30.8	42.7	42.6	31.2	35.6
Former (%)	32.1	20.3	33.6	36.2	27.8	29.7
Current ≤ 20 cigarettes/day (%)	18.3	39.2	21.7	16.2	27.6	27.5
Current > 20 cigarettes/day (%)	2.0	9.7	2.0	5.0	13.4	7.2
Physical activity (h/week)^d^	2.8 (0.1)	2.1 (0.3)	2.7 (0.3)	2.7 (0.0)	2.5 (0.1)	2.7 (0.1)
Alcohol intake from beverages (g/day)	14.7 (0.4)	11.0 (1.3)	13.9 (1.5)	19.8 (1.0)	19.1 (1.9)	19.7 (2.5)
Intake of lipid lowering medication (%)	4.1 (0.4)	2.6 (1.4)	2.2 (1.6)	2.7 (0.7)	4.6 (1.5)	7.5 (5.0)
Biomarkers						
Total cholesterol (mg/dL)	174.3 (0.8)	184.5 (2.6)	173.5 (3.1)	228.5 (1.9)	237.7 (3.6)	228.4 (13.2)
HDL-cholesterol (mg/dL)	47.8 (0.3)	44.8 (0.9)	47.6 (1.0)	59.7 (0.7)	52.1 (1.4)	63.4 (5.2)
LDL-cholesterol (mg/dL)^e^	104.1 (0.6)	113.2 (2.1)	103.0 (2.4)	155.2 (1.8)	167.5 (3.5)	148.9 (12.8)
Triglycerides (mg/dL)	113.4 (1.7)	133.6 (5.6)	113.3 (6.5)	153.6 (4.2)	205.7 (9.2)	182.4 (29.7)
hs-CRP (mg/dL)	0.17 (0.01)	0.24 (0.03)	0.30 (0.03)	0.19 (0.02)	0.26 (0.03)	0.21 (0.11)

^a^ Presented are age- and sex-adjusted mean (standard error) for continuous variables or percentages for categorical variables

^b^ Unadjusted mean (standard deviation) or percent

^c^ Age-adjusted mean (standard error)

^d^ Average of cycling and sports during summer and winter season

^e^ LDL-cholesterol was estimated using the Friedewald formula [22]

Of the 105 metabolites, three metabolites in EPIC-Potsdam and nine metabolites in EPIC-Heidelberg were associated with risk of stroke at *p* < 0.05 (Supplemental Tables 1 and 2). None of them remained associated after correction for multiple testing and none of them was overlapping in both studies. Therefore, the endpoint stroke was not further investigated.

**Fig. 2:**
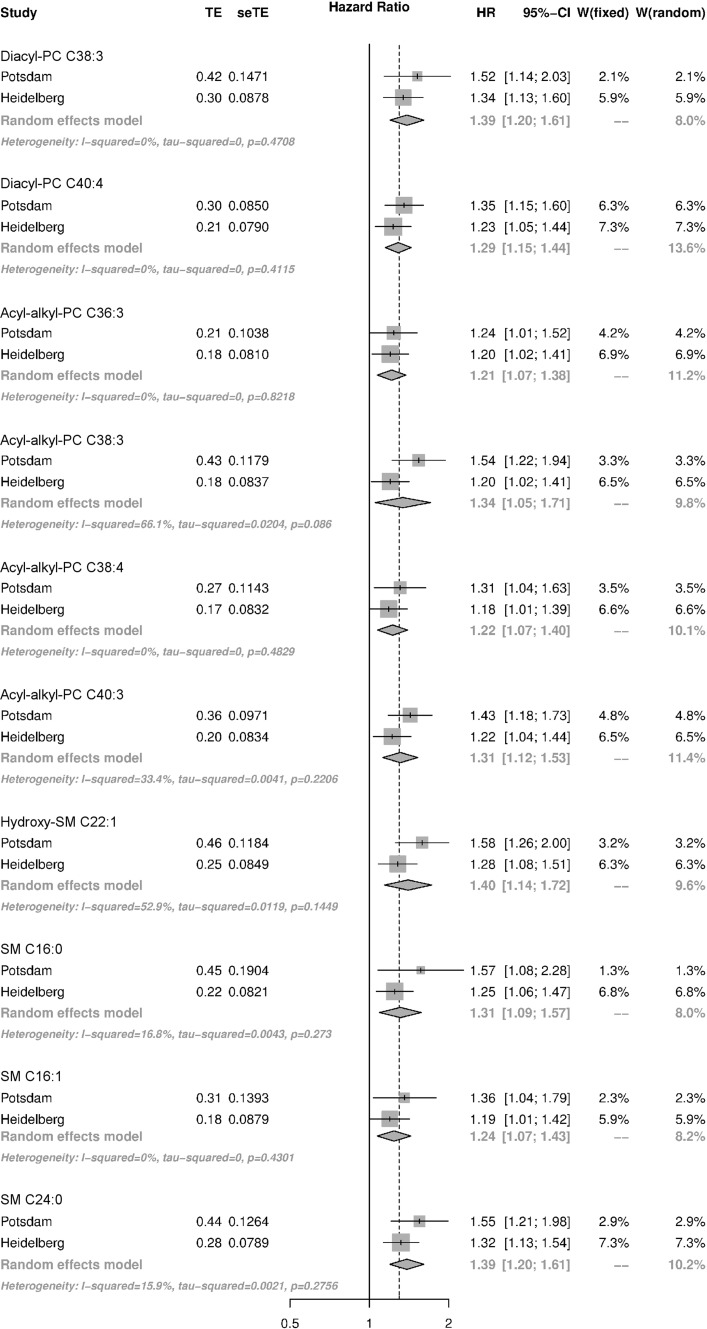
Forest plot of metabolites associated with risk of myocardial infarction (MI) in both study cohorts. Presented are hazard ratios (HR) and 95% confidence intervals for both study cohorts and pooled estimates from meta-analysis. HR were calculated in continuous models with standardized log2 transformed metabolite concentrations as exposure and incidence of MI as outcome. The model was stratified by age and adjusted for sex, alcohol intake, smoking, physical activity, education, fasting status, prevalent hypertension, BMI, and waist circumference. aa, diacyl; ae, acyl-alkyl; PC, phosphatidylcholine; seTE, standard error risk estimate; SM, sphingomyelin; TE, risk estimate (beta coefficient); W, study weight

Of all metabolites, 40 metabolites in EPIC-Potsdam and 15 metabolites in EPIC-Heidelberg were associated with risk of MI at *p* < 0.05 (Supplemental Tables 3 and 4). After correction for multiple testing, 19 metabolites remained. In both studies, ten metabolites were consistently associated with risk of MI, including diacyl-phosphatidylcholines C38:3 and C40:4; acyl-alkyl-phosphatidylcholines C36:3, C38:3, C38:4 and C40:3; as well as sphingomyelins C16:0, C24:0 and C16:1 and hydroxy-sphingomyelin C22:1 (Fig. 2). All of these metabolites were positively associated with risk of MI with pooled relative risks in the range of 1.21–1.40 per 1 SD increase in metabolite concentrations; and for all metabolites there was no heterogeneity between the two studies. In a sensitivity analysis, we found that the associations between sphingomyelins and MI risk were stronger for cases that occurred during the first 6 years of follow-up (Supplemental Table 5). We did not observe any gender-based differences.

**Fig. 3:**
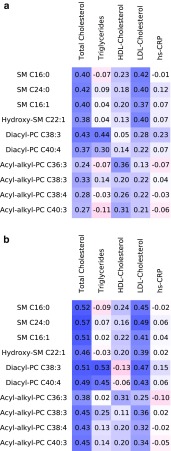
Correlation between metabolites associated with risk of myocardial infarction and established biomarkers of cardiovascular disease risk in the EPIC-Potsdam (**a**) and EPIC-Heidelberg subcohorts (**b**). Presented are Spearman partial correlation coefficients adjusted for age and sex. Blue color indicates positive correlation and red color inverse correlation. aa, diacyl; ae, acyl-alkyl; PC, phosphatidylcholine; SM, sphingomyelin

We next quantified the correlation of the ten identified metabolites with traditional CVD biomarkers and examined to what extent adjustment for these traditional CVD biomarkers affects the association between serum metabolites and risk of MI. All metabolites were positively correlated to total- and LDL-cholesterol with Spearman correlation coefficients in the range of 0.13–0.57 (Fig. 3). Diacyl-phosphatidylcholines C38:3 and C40:4 were positively correlated with triglycerides (Heidelberg r = 53 and r = 0.45; Potsdam: r = 0.44 and r = 0.30; respectively) and diacyl-phosphatidylcholine C38:3 with hs-CRP (Potsdam: r = 0.23; Heidelberg r = 015). Acyl-alkyl-phosphatidylcholines showed a positive correlation with HDL-cholesterol (r ranged from 0.11–0.36). After adjustment for LDL-cholesterol as well as total cholesterol the associations between sphingomyelins and acyl-alkyl-phosphatidylcholines and risk of MI were attenuated (Supplemental Table 6), whereas the associations remained for the diacyl-phosphatidylcholines. In contrast, additional adjustment for HDL-cholesterol, triglycerides or hs-CRP had minor impact on the associations between the metabolites and risk of MI. Adjusting for all CVD biomarkers simultaneously had a similar effect as adjusting for total and LDL-cholesterol; higher concentrations of diacyl-phosphatidylcholines C38:3 and C40:4, and in addition acyl-alkyl-phosphatidylcholine C36:3 remained associated with higher risk of MI in both study cohorts.

We studied these three metabolites in terms of risk prediction. To better understand their individual contribution, we first studied unadjusted models including one biomarker at a time (Table 2). Diacyl-phosphatidylcholine C38:3 performed best in discrimination with a C-statistic alone of 0.636 and 0.630 in Potsdam and Heidelberg; respectively; which was of higher magnitude than c-statistics alone of total cholesterol, triglycerides and hs-CRP in EPIC-Potsdam. Diacyl-phosphatidylcholine C40:4 had a C-statistic of 0.607 in EPIC-Potsdam and 0.619 in EPIC-Heidelberg, which was similar to total cholesterol; and acyl-alkyl-phosphatidylcholine C36:3 had the lowest c-statistic compared to all other biomarkers (EPIC-Potsdam: 0.507; EPIC-Heidelberg: 0.544; respectively). All three metabolites showed a good model calibration with Hosmer–Lemeshow *p* values larger than 0.05. When adding the metabolites to the basic adjustment model including established CVD risk factors the area under the ROC-curve could be improved from 0.826 (95% CI 0.798–0.854) to 0.828 (95% CI 0.800–0.857) in EPIC-Potsdam and from 0.824 (95% CI 0.791–0.858) to 0.832 (95% CI 0.799–0.865) in EPIC-Heidelberg (Supplemental Figure 1). In comparison to the model with classical CVD risk factors and established CVD biomarkers the areas under the ROC curves where further improved when adding the three metabolites [EPIC-Potsdam: from 0.836 (95% CI 0.810–0.863) to 0.838 (95% CI 0.811–0.866); EPIC-Heidelberg: from 0.834 (0.802–0.867) to 0.838 (95% CI 0.806–0.870)].

**Table 2:** Measures^a^ of discrimination and calibration to predict risk of myocardial infarction in EPIC-Potsdam and EPIC-Heidelberg for individual metabolites and biomarkers

Biomarker	Study population	C-statistic^b^	Hosmer–Lemeshow^c^	
			Χ^2^	*p* value
Acyl-alkyl-PC C36:3	Potsdam	0.507	15.15	0.056
	Heidelberg	0.544	9.91	0.271
Diacly-PC C38:3	Potsdam	0.636	8.92	0.349
	Heidelberg	0.630	10.23	0.249
Diacyl-PC C40:4	Potsdam	0.607	7.32	0.502
	Heidelberg	0.619	5.69	0.681
HDL	Potsdam	0.645	15.38	0.052
	Heidelberg	0.717	13.67	0.134
LDL	Potsdam	0.650	17.53	0.025
	Heidelberg	0.657	15.71	0.047
Total cholesterol	Potsdam	0.629	10.20	0.251
	Heidelberg	0.608	6.24	0.620
Triglycerides	Potsdam	0.628	19.55	0.012
	Heidelberg	0.706	13.62	0.092
hs-CRP	Potsdam	0.620	28.58	0.0002
	Heidelberg	0.675	33.77	< 0.0001

^a^ Presented are unadjusted models including one biomarker at a time. Better discrimination is mirrored by larger C-statistics and better calibration is indicated by Homer–Lemeshow smaller χ^2^ values and *p* value ≥ 0.05

^b^ Specifically, the c-statistic equals the area under the ROC curve, a measure of discrimination that mirrors the probability the model assigns a higher risk to future myocardial infarction cases compared to controls. It may range from 0.5 (no discrimination) to 1.0 (perfect discrimination) [23]

^c^ As a measure of model calibration, the Hosmer–Lemeshow statistic compares predicted and observed probabilities of myocardial infarction derived from deciles of predicted risk. Smaller χ^2^ values and larger *p* values specify better model fit. *P* values < 0.05 indicate difference between expected and observed probabilities [24]

*PC* phosphatidylcholine

## Discussion

The present study applied a targeted metabolomic approach to two cohorts of apparently healthy middle-aged adults who were followed on average over 7.5 years for incident CVD. Thereby, higher serum concentrations of four sphingomyelins and six phosphatidylcholines were linked to higher risk of MI independent of classical CVD risk factors. Of them diacyl-phosphatidylcholines C38:3 and C40:4, and acyl-alkyl-phosphatidylcholine C36:3 remained associated when additionally accounting for traditional CVD biomarkers in both study populations, and were also partly useful for CVD prediction. None of the studied metabolites were consistently associated with stroke risk.

A previous prospective study reported that alanine as well as medium and long-chain acylcarnitine levels predicted CVD events in an elderly high-risk population [25]. A prospective patient cohort found high concentrations of acylcarnitines, ketone-related metabolites and fatty acids and low concentrations of branched chain amino acids to be associated with higher risk of MI or death [3, 4]. Wang et al. [5] reported that dietary choline and gut microbiota metabolism of phosphatidylcholines promotes CVD events. These previous studies are not directly comparable to our study as they have been conducted either in CVD patients or in high-risk populations. In contrast, in our study we included originally healthy individuals and followed them over time until occurrence of a first incident CVD event. Würtz et al. [6] recently reported that higher phenylalanine concentrations were linked to higher CVD risk in population-based cohorts. This was not observed in our study. However, we previously observed that higher phenylalanine levels were linked to higher risk of type 2 diabetes in our population [11], which is a strong risk factor for CVD. For the present study we focused on individuals without a history of diabetes mellitus, which could be a reason for the discrepancy to the study by Würtz et al.

Recently, Ganna et al. [8] found 4 lipid metabolites including lyso-phosphatidylcholines and sphingomyelins that were linked to risk of coronary heart disease when investigating three population-based prospective cohorts. In addition, lipid metabolites, including three sphingomyelins and two phosphatidylcholines, were associated with risk of a composite CVD endpoint in the population-based Bruneck cohort [7]. In agreement, we found particularly higher concentrations of phosphatidylcholines and sphingomyelins linked to higher risk of MI. These metabolites have been previously suggested to be involved in the pathophysiologic process of atherosclerosis that often leads to the onset of CVD events. This process involves enzyme actions of sphingomyelinase and secretory phospholipase A2 that release free lipid species, such as fatty acids, lyso-phosphatidylcholines and ceramides, which may further rupture vessel walls [26–29]. In addition, these enzyme actions may cause severe modification of LDL-particles, and thereby promote inflammatory processes and ruptures at the vessel wall, which induce monocyte emigration, differentiation and foam cell formation, and may eventually result in atherogenic plaques and thrombosis. It has previously been observed that LDL-particles in atherogenic plaques were extensively enriched with sphingomyelins compared to plasma LDL-particles [30, 31]. In addition, oral administration of an inhibitor of sphingomyelin de-novo biosynthesis prevented atherosclerosis in apo-E knockout mice [27].

In a randomized controlled trial it was observed that treatment of CVD patients with statins led to lower plasma concentrations of sphingomyelins, including C16:0 and C24:0 [26]. This is in line with our observation that particularly sphingomyelins were linked to total cholesterol, and that adjustment for cholesterol levels attenuated the associations between two sphingomyelins and risk of MI. Previous cross-sectional studies reported that plasma concentrations of sphingomyelins were associated with subclinical atherosclerosis and coronary artery disease [32, 33]. In addition, in a small cohort of patients with acute coronary syndromes higher plasma sphingomyelin levels were linked to a worse prognosis [34]. However, the Multi Ethnic study did not find an association between total sphingomyelins and risk of coronary heart disease [35]. The present study identified several sphingomyelins that were positively associated with risk of MI in two cohorts, and it thus provides evidence for a prospective association. High sphingomyelin concentrations were particularly associated with high incidence of MI within the first 6 years of follow-up in the present study. These results support the hypothesis that elevation of serum sphingomyelin concentrations is linked to atherosclerosis, which may trigger the onset of MI.

**Fig. 4:**
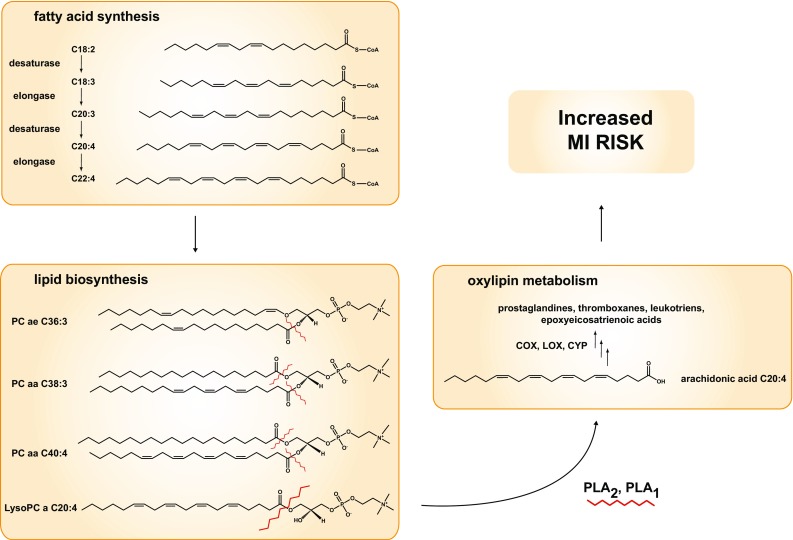
Schematic of the possible pathways of the association of acyl-alkyl-phosphatidylcholine C36:3 and diacyl-phosphatidylcholines C38:3 and C40:4 with risk of myocardial infarction. Fatty acid synthesis involves enzymatic reactions catalyzed by desaturases and elongases resulting in different chain length (e.g. C18) of different desaturations (e.g. C18:2) along it. These fatty aids are used in lipid biosynthesis and the same chains may appear in different molecules. Some specific lipids like the acyl-alkyl phosphatidylcholines (e.g. PC ae C36:3), diacyl phosphatidylcholines (PC aa C38:3) or lysophosphatidylcholines (LysoPC a C20:4) are associated with MI. In the following processes the fatty acids might be released from lipids by the activities of phospholipases PLA_2_ or PLA_1_ (cleavage sites is depicted by zigzag line). In further steps some of fatty acids such as arachidonic acid (C20:4) are metabolized to oxilipins (eicosanoids) by cyclooxygenases, lipooxygenases or cytochrome P450 monooxygenases (COX, LOX, CYP respectively) to prostaglandins, thromboxanes, leukotriens, or epoxyeicosatrienoic acids mediating inflammatory processes

In the present study, diacyl-phosphatidylcholines C38:3 and C40:4 as well as acyl-alkyl-phosphatidylcholine C36:3 remained associated with risk of MI when accounting for classical CVD risk factors as well as biomarkers. They were also partly useful for CVD prediction, particularly diacyl-phosphatidylcholine C38:3 which showed better discrimination than total cholesterol, triglycerides and CRP in EPIC-Potsdam. The identified metabolites have been previously associated with risk of type 2 diabetes in the EPIC-Potsdam cohort [11]. The three metabolites contain fatty acids that are interlinked via desaturase and elongase reactions (see Fig. 4). They may contain arachidonic acid as fatty acid residue which is an omega-6 fatty acid and can be released from the phospholipid by the enzymes phospholipase A1 and A2. Arachidonic acid is a precursor essential for eicosanoid biosynthesis such as prostaglandins and thromboxanes which are inflammatory mediators with various functions on the vascular system, which could be a possible mechanism for their positive association with risk of MI [36].

The null results that we observed for stroke may suggest that the serum concentrations of the metabolites measured in our study do not play a major role in the pathophysiology of stroke risk. Metabolic changes after an acute stroke event have previously been related to one-carbon-cycle metabolism, anaerobic glycolysis and hyper-homocysteinemia [37, 38]. In a recent investigation, low concentrations of lyso-phosphatidylcholines predicted stroke recurrence in TIA patients [39]. In our EPIC-Heidelberg study population, higher concentrations of three lyso-phosphatidylcholines were also linked to lower risk of stroke; however, this result was no longer observed after multiple testing corrections and was not consistent in EPIC-Potsdam. Thus, it has to be interpreted with caution. A number of neuro-protective properties have been suggested for lyso-phosphatidylcholines in vivo and in vitro, such as that they can serve as a suppressant for lipoprotein associated phospholipase A2 and thereby reduce its neuroinflammative properties [39–42]. It has also been reported that lyso-phsophatidylcholine levels increase in the brain in response to an acute stroke event, to mediate phagocyte recruitment; this could lead to reduced plasma levels of lyso-phosphatidylcholines [43, 44]. It is likely that metabolite concentrations change rapidly in response to an acute stroke event but this does not necessarily imply that they were altered years before disease onset. Future prospective studies are needed for further in depths investigation of the prospective association between metabolites and stroke risk.

### Strengths and limitations

Strengths of our study include that we conducted metabolomic measurements covering more than one hundred metabolites in two large prospective cohort studies that were well-phenotyped and followed over time for incidence of CVD. As we used two cohorts, we were able to directly replicate the results. We measured metabolite concentrations in blood samples of originally healthy adults and followed them until occurrence of a first CVD event, whereas previous studies were focused on high-risk populations. In addition, we investigated risk of MI and stroke separately.

However, the present study also had some limitations. We were limited to study only those metabolites that were included in the kit, and therefore might have missed associations of other metabolite classes. To address this limitation, targeted metabolomic studies that focus on other metabolites and untargeted metabolomic studies with no a priori assumptions should be conducted in the future. Furthermore, we obtained only a single blood sample at baseline in our studies, and metabolite concentrations may change over time. However, in a previous study we found a relatively high reliability of most of the metabolites included in our study over 4 month and others reported a high reliability over a 2-year period [45, 46]. Due to logistic reasons, participants of the EPIC-study did not necessarily provide fasting blood samples. We addressed this issue by adjusting for fasting status. In this study, we did not look at the inter-correlation of metabolites. This part has already been investigated in two previous studies by the authors by applying principal component analysis [11] and network analysis [47] to the same study population. As this was an observational study we cannot prove causality of the associations. However, we used a prospective design that addresses the issue of temporality of associations and we reproduced the results in different populations. Still, the possibility of reverse causality needs to be considered. We tried to account for this by stratifying the analysis by follow-up intervals.

## Conclusions

In summary, the present study identified novel candidates of sphingomyelin and phosphatidylcholine classes that were positively associated with risk of MI in healthy adults in two prospective cohorts. Of them three metabolites, that are involved in the arachidonic acid pathway, namely diacyl-phosphatidylcholines C38:3, C40:4 and acyl-alkyl-phosphatidylcholine C36:3, were associated with MI risk independent of traditional CVD risk factors and biomarkers, and were partly useful for CVD prediction. In contrast, we found no association between serum metabolites and risk of stroke. Based on their correlations with traditional CVD biomarkers, the identified metabolites point towards pathways of atherosclerosis and dyslipidaemia; and we particularly highlight the arachidonic acid pathway; however, future studies are needed to better understand these biological mechanisms.

## Electronic supplementary material

Below is the link to the electronic supplementary material.

Supplementary material 1 (DOC 599 kb)

Supplementary material 2 (PDF 22 kb)

Supplementary material 3 (PDF 19 kb)

## References

[1] Lewis GD, Asnani A, Gerszten RE (2008). Application of metabolomics to cardiovascular biomarker and pathway discovery. J Am Coll Cardiol.

[2] Gowda GA, Zhang S, Gu H, Asiago V, Shanaiah N, Raftery D (2008). Metabolomics-based methods for early disease diagnostics. Expert Rev Mol Diagn..

[3] Shah AA, Craig DM, Sebek JK (2012). Metabolic profiles predict adverse events after coronary artery bypass grafting. J Thorac Cardiovasc Surg.

[4] Shah SH, Sun JL, Stevens RD (2012). Baseline metabolomic profiles predict cardiovascular events in patients at risk for coronary artery disease. Am Heart J.

[5] Wang Z, Klipfell E, Bennett BJ (2011). Gut flora metabolism of phosphatidylcholine promotes cardiovascular disease. Nature.

[6] Wurtz P, Havulinna AS, Soininen P (2015). Metabolite profiling and cardiovascular event risk: a prospective study of 3 population-based cohorts. Circulation.

[7] Stegemann C, Pechlaner R, Willeit P (2014). Lipidomics profiling and risk of cardiovascular disease in the prospective population-based Bruneck study. Circulation.

[8] Ganna A, Salihovic S, Sundstrom J (2014). Large-scale metabolomic profiling identifies novel biomarkers for incident coronary heart disease. PLoS Genet.

[9] Boeing H, Wahrendorf J, Becker N (1999). EPIC-Germany—A source for studies into diet and risk of chronic diseases. European investigation into cancer and nutrition. Ann Nutr Metab.

[10] InterAct C, Langenberg C, Sharp S (2011). Design and cohort description of the InterAct Project: an examination of the interaction of genetic and lifestyle factors on the incidence of type 2 diabetes in the EPIC Study. Diabetologia.

[11] Floegel A, Stefan N, Yu Z (2013). Identification of serum metabolites associated with risk of type 2 diabetes using a targeted metabolomic approach. Diabetes.

[12] Bergmann MM, Bussas U, Boeing H (1999). Follow-up procedures in EPIC-Germany–data quality aspects. European Prospective Investigation into Cancer and Nutrition. Ann Nutr Metab.

[13] Schienkiewitz A, Schulze MB, Hoffmann K, Kroke A, Boeing H (2006). Body mass index history and risk of type 2 diabetes: results from the European Prospective Investigation into Cancer and Nutrition (EPIC)-Potsdam Study. Am J Clin Nutr.

[14] Romisch-Margl W, Prehn C, Bogumil R, Rohring C, Suhre K, Adamski J (2012). Procedure for tissue sample preparation and metabolite extraction for high-throughput targeted metabolomics. Metabolomics.

[15] Zukunft S, Sorgenfrei M, Prehn C, Möller G, Adamski J (2013). Targeted metabolomics of dried blood spot extracts. Chromatographia.

[16] Kuhn T, Floegel A, Sookthai D (2016). Higher plasma levels of lysophosphatidylcholine 18:0 are related to a lower risk of common cancers in a prospective metabolomics study. BMC Med.

[17] Lazar C, Meganck S, Taminau J (2013). Batch effect removal methods for microarray gene expression data integration: a survey. Brief Bioinform.

[18] Hubert M, Engelen S (2004). Robust PCA and classification in biosciences. Bioinformatics.

[19] Prentice RL (1995). Design issues in cohort studies. Stat Methods Med Res.

[20] Benjamini Y, Hochberg Y (1995). Controlling the false discovery rate—a practical and powerful approach to multiple testing. J R Stat Soc B Met.

[21] Hardy RJ, Thompson SG (1998). Detecting and describing heterogeneity in meta-analysis. Stat Med.

[22] Friedewald WT, Levy RI, Fredrickson DS (1972). Estimation of the concentration of low-density lipoprotein cholesterol in plasma, without use of the preparative ultracentrifuge. Clin Chem.

[23] Hanley JA, McNeil BJ (1982). The meaning and use of the area under a receiver operating characteristic (ROC) curve. Radiology.

[24] Hosmer DW, Lemeshow S (2000). Applied logistic regression.

[25] Rizza S, Copetti M, Rossi C (2014). Metabolomics signature improves the prediction of cardiovascular events in elderly subjects. Atherosclerosis.

[26] Bergheanu SC, Reijmers T, Zwinderman AH (2008). Lipidomic approach to evaluate rosuvastatin and atorvastatin at various dosages: investigating differential effects among statins. Curr Med Res Opin.

[27] Park TS, Panek RL, Rekhter MD (2006). Modulation of lipoprotein metabolism by inhibition of sphingomyelin synthesis in ApoE knockout mice. Atherosclerosis.

[28] Oestvang J, Bonnefont-Rousselot D, Ninio E, Hakala JK, Johansen B, Anthonsen MW (2004). Modification of LDL with human secretory phospholipase A(2) or sphingomyelinase promotes its arachidonic acid-releasing propensity. J Lipid Res.

[29] Bismuth J, Lin P, Yao Q, Chen C (2008). Ceramide: A common pathway for atherosclerosis?. Atherosclerosis.

[30] Hoff HF, Morton RE (1985). Lipoproteins containing apo B extracted from human aortas. Structure and function. Ann N Y Acad Sci.

[31] Jeong T, Schissel SL, Tabas I, Pownall HJ, Tall AR, Jiang X (1998). Increased sphingomyelin content of plasma lipoproteins in apolipoprotein E knockout mice reflects combined production and catabolic defects and enhances reactivity with mammalian sphingomyelinase. J Clin Invest.

[32] Nelson JC, Jiang XC, Tabas I, Tall A, Shea S (2006). Plasma sphingomyelin and subclinical atherosclerosis: findings from the multi-ethnic study of atherosclerosis. Am J Epidemiol.

[33] Jiang XC, Paultre F, Pearson TA (2000). Plasma sphingomyelin level as a risk factor for coronary artery disease. Arterioscler Thromb Vasc Biol.

[34] Schlitt A, Blankenberg S, Yan D (2006). Further evaluation of plasma sphingomyelin levels as a risk factor for coronary artery disease. Nutr Metab (Lond).

[35] Yeboah J, McNamara C, Jiang XC (2010). Association of plasma sphingomyelin levels and incident coronary heart disease events in an adult population: multi-Ethnic Study of Atherosclerosis. Arterioscler Thromb Vasc Biol.

[36] Goodman DS (1987). The role of arachidonic acid metabolites in cardiovascular homeostasis. Biochemical, histological and clinical cardiovascular effects of non-steroidal anti-inflammatory drugs and their interactions with cardiovascular drugs. Drugs.

[37] Jiang Z, Sun J, Liang Q (2011). A metabonomic approach applied to predict patients with cerebral infarction. Talanta.

[38] Jung JY, Lee HS, Kang DG (2011). 1H-NMR-based metabolomics study of cerebral infarction. Stroke.

[39] Jove M, Mauri-Capdevila G, Suarez I (2015). Metabolomics predicts stroke recurrence after transient ischemic attack. Neurology.

[40] Cunningham TJ, Yao L, Lucena A (2008). Product inhibition of secreted phospholipase A2 may explain lysophosphatidylcholines’ unexpected therapeutic properties. J Inflamm (Lond).

[41] Pinto F, Brenner T, Dan P, Krimsky M, Yedgar S (2003). Extracellular phospholipase A2 inhibitors suppress central nervous system inflammation. Glia.

[42] Blondeau N, Lauritzen I, Widmann C, Lazdunski M, Heurteaux C (2002). A potent protective role of lysophospholipids against global cerebral ischemia and glutamate excitotoxicity in neuronal cultures. J Cereb Blood Flow Metab.

[43] Koizumi S, Yamamoto S, Hayasaka T (2010). Imaging mass spectrometry revealed the production of lyso-phosphatidylcholine in the injured ischemic rat brain. Neuroscience.

[44] Zhang Z, Lee YC, Kim SJ (2007). Production of lysophosphatidylcholine by cPLA2 in the brain of mice lacking PPT1 is a signal for phagocyte infiltration. Hum Mol Genet.

[45] Floegel A, Drogan D, Wang-Sattler R (2011). Reliability of serum metabolite concentrations over a 4-month period using a targeted metabolomic approach. PLoS ONE.

[46] Carayol M, Licaj I, Achaintre D (2015). Reliability of serum metabolites over a two-year period: a targeted metabolomic approach in fasting and non-fasting samples from EPIC. PLoS ONE.

[47] Floegel A, Wientzek A, Bachlechner U (2014). Linking diet, physical activity, cardiorespiratory fitness and obesity to serum metabolite networks: findings from a population-based study. Int J Obes (Lond).

