# Supplementary material for: Serum metabolites and risk of myocardial infarction and ischemic stroke: a targeted metabolomic approach in two German prospective cohorts
Source: Eur J Epidemiol. 2017 Nov 27;33(1):55–66. doi: 10.1007/s10654-017-0333-0 (PMC5803284; doi:10.1007/s10654-017-0333-0)
Supplement: Supplementary file 1 — Supplementary material 1 (DOC 599 kb) [file 10654_2017_333_MOESM1_ESM.doc]

**Supplementary Material**

**Serum metabolites and risk of myocardial infarction and ischemic stroke: a targeted metabolomic approach in two German prospective cohorts**

**Anna Floegel, Tilman Kühn, Disorn Sookthai, Theron Johnson, Cornelia Prehn, Ulrike Rolle-Kampczyk, Wolfgang Otto, Cornelia Weikert, Thomas Illig, Martin von Bergen, Jerzy Adamski, Heiner Boeing, Rudolf Kaaks, and Tobias Pischon.**

**Supplementary Methods**

Study population

The European Prospective Investigation into Cancer and Nutrition (EPIC)-Germany study is part of the EPIC study, a prospective cohort study which is conducted in multiple centers across 10 European countries with the main objective to investigate diet and risk of cancer and other chronic diseases. The German part of the EPIC study includes two study centers, located in Potsdam, Brandenburg (North-East-Germany), and Heidelberg, Baden-Württemberg (South-West-Germany). Participants mainly aged between 35 and 65 years were recruited from the general adult population in the two cities and surrounding areas with response rates of 22.7% and 38.3% in Potsdam and Heidelberg, respectively . A total of 27548 people from Potsdam and 25540 people from Heidelberg (overall 57% women) consented to participate and took part in the baseline examination between 1994 and 1998. Thereby, participants underwent anthropometric and blood pressure measurements, provided a PC-guided interview on medical history and lifestyle factors, and filled in a self-administered questionnaire on sociodemographic and lifestyle factors as well as a food frequency questionnaire. In addition, they provided a blood sample that was immediately processed by trained staff following standardized protocols, fractionated into plasma, serum, buffy coat, erythrocytes and blood clot and ever-since stored in large tanks of liquid nitrogen at -196°C . Incident cases of chronic diseases, including MI and stroke, were identified every two to three years by combination of active and passive follow-up procedures. In cases of multiple incident events we considered only the first event for the current analysis. The studies were approved by the ethics committees of the Medical Society of the State of Brandenburg and the Medical Faculty of the University of Heidelberg.

Metabolomic measurements

Metabolomic analyses were conducted at the Genome Analysis Center, Helmholtz Zentrum München (Potsdam samples), and at the Department of Metabolomics, Helmholtz Centre for Environmental Research, Leipzig (Heidelberg samples), using the Absolute*IDQ*^TM^ p150 and p180 kits (BIOCRATES Life Sciences AG, Innsbruck, Austria); respectively. The following steps were conducted: After centrifugation, 10 µl of serum were inserted into a filter on a 96-well sandwich plate with stable isotope-labelled internal standards. Amino acids were derivated with 5% phenylisothiocyanate reagent. Next, 5 mM ammonium acetate in methanol was used to extract metabolites and internal standards. After centrifugation of the solution through a filter membrane it was diluted with MS running solvent. The finally extracted samples were analysed by flow injection analysis tandem mass spectrometry (FIA-MS/MS) and liquid chromatography (LC)-MS/MS, and metabolites were quantified in µmol/L using internal standards. The method has been validated by the manufacturer in agreement with the FDA guideline “Guidance for industry – bioanalytical method validation, May 2001” . Analytical specifications have been described in detail previously, and median coefficients of variation were 7.3% within plate and 11.3% between plates for our samples . Samples were randomly analysed in respect of case-status, to account for run-order effects.

**Supplemental Table 1: Selection of serum metabolites associated with risk of stroke in EPIC-Potsdam (n=2525)**

| **Rank^a^** | **Metabolite** | **Hazard Ratio^b^** | **95% CI** | **Raw p-value** | **FDR p-value** |
| --- | --- | --- | --- | --- | --- |
| **1** | DIACYL-PC C42:0 | 0.82 | (0.69, 0.97) | 0.02104236540 | 1 |
| **2** | Glycine | 0.91 | (0.83, 0.99) | 0.03336635740 | 1 |
| **3** | Proline | 0.91 | (0.83, 1) | 0.04383545590 | 1 |
| **4** | DIACYL-PC C32:3 | 1.16 | (0.98, 1.38) | 0.08772477430 | 1 |
| **5** | ACYL-ALKYL-PC C44:6 | 0.86 | (0.72, 1.03) | 0.09362422290 | 1 |
| **6** | ACYL-ALKYL-PC C42:1 | 1.14 | (0.96, 1.37) | 0.13990664070 | 1 |
| **7** | ACYL-ALKYL-PC C36:3 | 1.23 | (0.93, 1.63) | 0.15274600520 | 1 |
| **8** | DIACYL-PC C34:4 | 1.15 | (0.94, 1.4) | 0.16690580040 | 1 |
| **9** | Ornithine | 0.93 | (0.83, 1.03) | 0.16757388650 | 1 |
| **10** | LYSO-PC C17:0 | 0.89 | (0.74, 1.05) | 0.17011691660 | 1 |
| **11** | DIACYL-PC C32:1 | 1.15 | (0.94, 1.4) | 0.17115951040 | 1 |
| **12** | DIACYL-PC C32:2 | 1.15 | (0.94, 1.41) | 0.17248915420 | 1 |
| **13** | ACYL-ALKYL-PC C42:5 | 0.89 | (0.76, 1.05) | 0.17602412710 | 1 |
| **14** | DIACYL-PC C42:4 | 1.12 | (0.95, 1.33) | 0.17897109720 | 1 |
| **15** | DIACYL-PC C34:3 | 1.19 | (0.92, 1.52) | 0.18145677860 | 1 |
| **16** | DIACYL-PC C42:1 | 0.88 | (0.73, 1.06) | 0.18954680640 | 1 |
| **17** | Threonine | 0.92 | (0.82, 1.04) | 0.19427125130 | 1 |
| **18** | Glutamine | 0.94 | (0.86, 1.03) | 0.20087451030 | 1 |
| **19** | ACYL-ALKYL-PC C34:2 | 1.20 | (0.91, 1.58) | 0.20097013010 | 1 |
| **20** | ACYL-ALKYL-PC C44:5 | 0.90 | (0.76, 1.07) | 0.22382794420 | 1 |
| **21** | LYSO-PC C16:1 | 1.11 | (0.94, 1.31) | 0.23631821670 | 1 |
| **22** | DIACYL-PC C42:2 | 0.90 | (0.77, 1.07) | 0.23697877710 | 1 |
| **23** | ACYL-ALKYL-PC C40:6 | 0.91 | (0.78, 1.06) | 0.23754066060 | 1 |
| **24** | ACYL-ALKYL-PC C36:0 | 1.09 | (0.93, 1.27) | 0.27233525350 | 1 |
| **25** | ACYL-ALKYL-PC C42:4 | 0.91 | (0.76, 1.09) | 0.28368543390 | 1 |
| **26** | DIACYL-PC C40:4 | 1.11 | (0.92, 1.35) | 0.28508127610 | 1 |
| **27** | LYSO-PC C18:2 | 0.92 | (0.79, 1.07) | 0.28539718880 | 1 |
| **28** | LYSO-PC C18:0 | 0.94 | (0.82, 1.06) | 0.31250479720 | 1 |
| **29** | Carnitine | 0.93 | (0.8, 1.07) | 0.31781011710 | 1 |
| **30** | ACYL-ALKYL-PC C30:0 | 0.91 | (0.76, 1.09) | 0.31914583990 | 1 |
| **31** | ACYL-ALKYL-PC C34:3 | 1.13 | (0.89, 1.43) | 0.32167762190 | 1 |
| **32** | Tryptophan | 0.95 | (0.86, 1.06) | 0.33849038230 | 1 |
| **33** | LYSO-PC C20:3 | 1.10 | (0.91, 1.33) | 0.34093863680 | 1 |
| **34** | DIACYL-PC C36:0 | 0.93 | (0.78, 1.1) | 0.38302805950 | 1 |
| **35** | DIACYL-PC C42:5 | 1.07 | (0.92, 1.26) | 0.38412209440 | 1 |
| **36** | ACYL-ALKYL-PC C38:3 | 1.11 | (0.87, 1.41) | 0.39173255210 | 1 |
| **37** | ACYL-ALKYL-PC C44:4 | 0.92 | (0.76, 1.12) | 0.41752735290 | 1 |
| **38** | Serine | 0.95 | (0.84, 1.08) | 0.41810269360 | 1 |
| **39** | ACYL-ALKYL-PC C36:4 | 1.10 | (0.86, 1.41) | 0.43354819890 | 1 |
| **40** | HYDROXY-SM C14:1 | 0.94 | (0.8, 1.1) | 0.43480355620 | 1 |
| **41** | HYDROXY-SM C16:1 | 0.94 | (0.79, 1.11) | 0.44021826730 | 1 |
| **42** | DIACYL-PC C32:0 | 1.11 | (0.85, 1.44) | 0.45051976150 | 1 |
| **43** | DIACYL-PC C40:3 | 1.06 | (0.91, 1.22) | 0.47096617990 | 1 |
| **44** | DIACYL-PC C30:0 | 1.07 | (0.88, 1.31) | 0.48414314370 | 1 |
| **45** | ACYL-ALKYL-PC C40:5 | 0.95 | (0.81, 1.11) | 0.50328045030 | 1 |
| **46** | ACYL-ALKYL-PC C38:1 | 1.06 | (0.89, 1.27) | 0.52579745920 | 1 |
| **47** | ACYL-ALKYL-PC C36:1 | 1.08 | (0.85, 1.36) | 0.52667646310 | 1 |
| **48** | Histidine | 0.96 | (0.84, 1.09) | 0.52677743930 | 1 |
| **49** | SM C20:2 | 1.05 | (0.9, 1.23) | 0.52871099690 | 1 |
| **50** | ACYL-ALKYL-PC C38:2 | 1.06 | (0.89, 1.26) | 0.53850606310 | 1 |
| **51** | DIACYL-PC C38:3 | 1.10 | (0.81, 1.51) | 0.54280740460 | 1 |
| **52** | DIACYL-PC C36:3 | 1.17 | (0.71, 1.92) | 0.54467462880 | 1 |
| **53** | HYDROXY-SM C22:2 | 0.95 | (0.81, 1.12) | 0.54590162150 | 1 |
| **54** | Arginine | 0.96 | (0.83, 1.1) | 0.54610250320 | 1 |
| **55** | DIACYL-PC C36:1 | 1.09 | (0.82, 1.44) | 0.56459158090 | 1 |
| **56** | DIACYL-PC C34:1 | 1.13 | (0.74, 1.74) | 0.57273583350 | 1 |
| **57** | ACYL-ALKYL-PC C42:3 | 0.96 | (0.81, 1.13) | 0.60067589240 | 1 |
| **58** | Isoleucine | 1.09 | (0.79, 1.5) | 0.60141608740 | 1 |
| **59** | ACYL-ALKYL-PC C34:0 | 1.05 | (0.87, 1.26) | 0.60311724440 | 1 |
| **60** | ACYL-ALKYL-PC C40:4 | 0.96 | (0.81, 1.13) | 0.61809779530 | 1 |
| **61** | DIACYL-PC C36:4 | 1.11 | (0.74, 1.66) | 0.62058458550 | 1 |
| **62** | ACYL-ALKYL-PC C34:1 | 1.05 | (0.84, 1.32) | 0.65773153740 | 1 |
| **63** | DIACYL-PC C28:1 | 1.05 | (0.85, 1.28) | 0.66324747710 | 1 |
| **64** | SM C24:0 | 1.05 | (0.84, 1.3) | 0.69260969370 | 1 |
| **65** | SM C16:1 | 1.05 | (0.81, 1.36) | 0.69653379850 | 1 |
| **66** | Valine | 1.07 | (0.74, 1.55) | 0.70440268220 | 1 |
| **67** | DIACYL-PC C40:2 | 1.03 | (0.87, 1.22) | 0.75116826270 | 1 |
| **68** | ACYL-ALKYL-PC C44:3 | 0.97 | (0.82, 1.15) | 0.75532575230 | 1 |
| **69** | SM C18:1 | 1.03 | (0.83, 1.28) | 0.75727100770 | 1 |
| **70** | DIACYL-PC C38:4 | 1.04 | (0.81, 1.33) | 0.75816390600 | 1 |
| **71** | DIACYL-PC C38:0 | 0.97 | (0.82, 1.16) | 0.77015009440 | 1 |
| **72** | DIACYL-PC C40:6 | 0.98 | (0.82, 1.16) | 0.77593019220 | 1 |
| **73** | DIACYL-PC C36:5 | 0.98 | (0.81, 1.17) | 0.78563246270 | 1 |
| **74** | ACYL-ALKYL-PC C32:2 | 1.02 | (0.86, 1.21) | 0.80409704300 | 1 |
| **75** | DIACYL-PC C34:2 | 1.03 | (0.78, 1.37) | 0.81248831400 | 1 |
| **76** | Acetyl carnitine | 1.02 | (0.84, 1.24) | 0.81465324050 | 1 |
| **77** | ACYL-ALKYL-PC C36:2 | 1.02 | (0.83, 1.26) | 0.83894831330 | 1 |
| **78** | Tyrosine | 1.02 | (0.83, 1.24) | 0.85651121560 | 1 |
| **79** | ACYL-ALKYL-PC C40:1 | 0.98 | (0.83, 1.17) | 0.85778638000 | 1 |
| **80** | LYSO-PC C16:0 | 0.99 | (0.84, 1.16) | 0.86151352590 | 1 |
| **81** | Phenylalanine | 0.99 | (0.84, 1.16) | 0.86403901350 | 1 |
| **82** | ACYL-ALKYL-PC C36:5 | 1.02 | (0.84, 1.23) | 0.86516272370 | 1 |
| **83** | LYSO-PC C18:1 | 0.98 | (0.82, 1.18) | 0.86711970110 | 1 |
| **84** | ACYL-ALKYL-PC C42:2 | 0.99 | (0.85, 1.16) | 0.88552947720 | 1 |
| **85** | ACYL-ALKYL-PC C38:6 | 0.99 | (0.82, 1.18) | 0.88945197480 | 1 |
| **86** | LYSO-PC C20:4 | 1.01 | (0.84, 1.22) | 0.90653689880 | 1 |
| **87** | ACYL-ALKYL-PC C38:0 | 0.99 | (0.83, 1.18) | 0.92342239010 | 1 |
| **88** | HYDROXY-SM C24:1 | 0.99 | (0.83, 1.18) | 0.92477558300 | 1 |
| **89** | DIACYL-PC C36:2 | 1.01 | (0.82, 1.24) | 0.93268727860 | 1 |
| **90** | ACYL-ALKYL-PC C40:2 | 0.99 | (0.83, 1.18) | 0.93588337710 | 1 |
| **91** | DIACYL-PC C38:5 | 0.99 | (0.83, 1.19) | 0.94108423380 | 1 |
| **92** | DIACYL-PC C40:5 | 0.99 | (0.83, 1.18) | 0.94332407830 | 1 |
| **93** | ACYL-ALKYL-PC C40:3 | 1.00 | (0.84, 1.21) | 0.95796640740 | 1 |
| **94** | SM C24:1 | 1.00 | (0.84, 1.21) | 0.95973503310 | 1 |
| **95** | SM C16:0 | 1.00 | (0.82, 1.23) | 0.96104053450 | 1 |
| **96** | SM C18:0 | 1.00 | (0.83, 1.22) | 0.96523525370 | 1 |
| **97** | Hexose | 1.00 | (0.83, 1.2) | 0.96824536600 | 1 |
| **98** | DIACYL-PC C38:6 | 1.00 | (0.83, 1.22) | 0.96988275250 | 1 |
| **99** | ACYL-ALKYL-PC C38:4 | 1.00 | (0.83, 1.22) | 0.97318680050 | 1 |
| **100** | ACYL-ALKYL-PC C38:5 | 1.00 | (0.83, 1.2) | 0.98007660470 | 1 |
| **101** | HYDROXY-SM C22:1 | 1.00 | (0.83, 1.2) | 0.98766772480 | 1 |
| **102** | DIACYL-PC C36:6 | 1.00 | (0.83, 1.21) | 0.99605385650 | 1 |
| **103** | LYSO-PC C28:1 | 1.00 | (0.83, 1.21) | 0.99699383260 | 1 |
| **104** | ACYL-ALKYL-PC C30:2 | 1.00 | (0.82, 1.22) | 0.99915967700 | 1 |
| **105** | ACYL-ALKYL-PC C32:1 | 1.00 | (0.84, 1.19) | 0.99954948530 | 1 |

^a^Metabolites were ranked from lowest to highest raw p-value for the association between metabolites and risk of stroke.

^b^Hazard ratios were derived from Cox regression analysis and refer to standardized log2 transformed metabolite concentrations. The model was adjusted for age, sex, alcohol intake from beverages (non-consumers; women: >0-6 g/d, 6-12 g/d, >12 g/d; men: >0-12 g/d, 12-24 g/d, >24 g/d), smoking (never, former, current ≤20 cigarettes/d, current >20 cigarettes/d), physical activity (cycling and sports in h/week), education (no degree/vocational training; trade/technical school; university degree), prevalent hypertension (yes/no), BMI (kg/m²), waist circumference (cm) and fasting status.

Abbreviations: PC, phosphatidylcholine; SM, sphingomyelin.

**Supplemental Table 2: Selection of serum metabolites associated with risk of stroke in EPIC-Heidelberg (n=1115)**

| **Rank^a^** | **Metabolite** | **Hazard Ratio^b^** | **95% CI** | **Raw p-value** | **FDR p-value** |
| --- | --- | --- | --- | --- | --- |
| **1** | LYSO-PC C18:2 | 0.68 | (0.53, 0.86) | 0.00129913840 | 0.1364095294 |
| **2** | LYSO-PC C17:0 | 0.74 | (0.6, 0.92) | 0.00705952050 | 0.7412496532 |
| **3** | LYSO-PC C18:1 | 0.75 | (0.61, 0.93) | 0.00989482660 | 1 |
| **4** | DIACYL-PC C38:3 | 1.28 | (1.05, 1.58) | 0.01739352350 | 1 |
| **5** | DIACYL-PC C40:5 | 1.25 | (1.04, 1.52) | 0.01974764140 | 1 |
| **6** | DIACYL-PC C36:0 | 0.79 | (0.64, 0.98) | 0.02927685650 | 1 |
| **7** | Phenylalanine | 0.79 | (0.63, 0.98) | 0.03060509380 | 1 |
| **8** | Serine | 0.80 | (0.65, 0.98) | 0.03373110850 | 1 |
| **9** | DIACYL-PC C40:4 | 1.22 | (1.01, 1.47) | 0.03451521160 | 1 |
| **10** | ACYL-ALKYL-PC C38:2 | 0.82 | (0.66, 1) | 0.05501537310 | 1 |
| **11** | ACYL-ALKYL-PC C42:1 | 1.20 | (0.98, 1.47) | 0.08293677550 | 1 |
| **12** | ACYL-ALKYL-PC C36:4 | 1.19 | (0.97, 1.46) | 0.09080894890 | 1 |
| **13** | Tryptophan | 0.84 | (0.68, 1.03) | 0.09485681350 | 1 |
| **14** | DIACYL-PC C32:3 | 0.82 | (0.66, 1.04) | 0.09944252450 | 1 |
| **15** | DIACYL-PC C34:1 | 1.19 | (0.97, 1.47) | 0.10335771070 | 1 |
| **16** | ACYL-ALKYL-PC C38:1 | 0.86 | (0.71, 1.04) | 0.10928609320 | 1 |
| **17** | Carnitine | 0.83 | (0.66, 1.04) | 0.11171201960 | 1 |
| **18** | Tyrosine | 0.83 | (0.66, 1.06) | 0.12938475970 | 1 |
| **19** | DIACYL-PC C32:1 | 1.18 | (0.95, 1.46) | 0.13487197520 | 1 |
| **20** | ACYL-ALKYL-PC C42:4 | 1.17 | (0.95, 1.44) | 0.13997760680 | 1 |
| **21** | DIACYL-PC C38:4 | 1.16 | (0.95, 1.43) | 0.14785880040 | 1 |
| **22** | ACYL-ALKYL-PC C34:0 | 0.86 | (0.7, 1.06) | 0.15335075090 | 1 |
| **23** | ACYL-ALKYL-PC C36:2 | 0.81 | (0.61, 1.08) | 0.15386662870 | 1 |
| **24** | ACYL-ALKYL-PC C36:1 | 0.84 | (0.67, 1.07) | 0.15842660250 | 1 |
| **25** | ACYL-ALKYL-PC C32:2 | 0.85 | (0.68, 1.07) | 0.16570781660 | 1 |
| **26** | DIACYL-PC C38:0 | 0.85 | (0.68, 1.07) | 0.16655958670 | 1 |
| **27** | LYSO-PC C20:4 | 0.86 | (0.68, 1.07) | 0.17660259210 | 1 |
| **28** | Threonine | 1.16 | (0.93, 1.44) | 0.18411517570 | 1 |
| **29** | Arginine | 0.88 | (0.72, 1.07) | 0.20737985430 | 1 |
| **30** | DIACYL-PC C40:2 | 0.87 | (0.7, 1.08) | 0.21493385840 | 1 |
| **31** | DIACYL-PC C42:5 | 1.13 | (0.92, 1.38) | 0.23436023410 | 1 |
| **32** | ACYL-ALKYL-PC C38:0 | 0.87 | (0.69, 1.09) | 0.23632887910 | 1 |
| **33** | HYDROXY-SM C22:2 | 0.87 | (0.69, 1.1) | 0.23887308010 | 1 |
| **34** | ACYL-ALKYL-PC C38:4 | 1.12 | (0.93, 1.34) | 0.23924515720 | 1 |
| **35** | DIACYL-PC C40:3 | 1.12 | (0.92, 1.36) | 0.25694476740 | 1 |
| **36** | DIACYL-PC C36:3 | 1.12 | (0.92, 1.38) | 0.26007134920 | 1 |
| **37** | DIACYL-PC C36:4 | 1.13 | (0.91, 1.42) | 0.27570195920 | 1 |
| **38** | HYDROXY-SM C14:1 | 0.88 | (0.69, 1.11) | 0.27607476430 | 1 |
| **39** | Glutamine | 0.88 | (0.7, 1.12) | 0.29318767500 | 1 |
| **40** | ACYL-ALKYL-PC C42:2 | 1.11 | (0.91, 1.35) | 0.29914088270 | 1 |
| **41** | HYDROXY-SM C24:1 | 0.90 | (0.73, 1.11) | 0.31608056270 | 1 |
| **42** | Hexose | 1.15 | (0.87, 1.53) | 0.32025073640 | 1 |
| **43** | ACYL-ALKYL-PC C42:3 | 0.89 | (0.71, 1.12) | 0.32302475220 | 1 |
| **44** | DIACYL-PC C28:1 | 0.89 | (0.71, 1.12) | 0.33267771890 | 1 |
| **45** | LYSO-PC C20:3 | 0.90 | (0.73, 1.11) | 0.33894593470 | 1 |
| **46** | Proline | 0.90 | (0.72, 1.12) | 0.34414096780 | 1 |
| **47** | LYSO-PC C18:0 | 0.91 | (0.74, 1.11) | 0.34431077580 | 1 |
| **48** | DIACYL-PC C36:6 | 0.90 | (0.73, 1.12) | 0.34985929320 | 1 |
| **49** | Ornithine | 0.90 | (0.72, 1.12) | 0.35106166550 | 1 |
| **50** | LYSO-PC C16:1 | 0.91 | (0.74, 1.12) | 0.35331949510 | 1 |
| **51** | ACYL-ALKYL-PC C40:2 | 0.90 | (0.72, 1.13) | 0.36138683440 | 1 |
| **52** | SM C18:0 | 1.10 | (0.9, 1.35) | 0.36627084960 | 1 |
| **53** | SM C24:0 | 0.91 | (0.75, 1.12) | 0.37697444690 | 1 |
| **54** | DIACYL-PC C36:1 | 1.10 | (0.89, 1.34) | 0.37827790300 | 1 |
| **55** | ACYL-ALKYL-PC C32:1 | 0.91 | (0.74, 1.12) | 0.37961889620 | 1 |
| **56** | ACYL-ALKYL-PC C40:6 | 0.90 | (0.72, 1.13) | 0.37986197020 | 1 |
| **57** | HYDROXY-SM C16:1 | 0.91 | (0.72, 1.13) | 0.38227633690 | 1 |
| **58** | DIACYL-PC C36:2 | 1.09 | (0.88, 1.36) | 0.41829849380 | 1 |
| **59** | ACYL-ALKYL-PC C34:3 | 0.92 | (0.75, 1.13) | 0.43517807560 | 1 |
| **60** | LYSO-PC C28:1 | 0.91 | (0.73, 1.15) | 0.44865926400 | 1 |
| **61** | DIACYL-PC C36:5 | 0.92 | (0.75, 1.14) | 0.45471922640 | 1 |
| **62** | ACYL-ALKYL-PC C36:5 | 1.08 | (0.88, 1.32) | 0.46000745620 | 1 |
| **63** | Acetyl carnitine | 1.09 | (0.87, 1.36) | 0.47332349030 | 1 |
| **64** | Histidine | 0.92 | (0.73, 1.16) | 0.48360325100 | 1 |
| **65** | Isoleucine | 0.92 | (0.72, 1.17) | 0.48900196130 | 1 |
| **66** | ACYL-ALKYL-PC C40:4 | 1.07 | (0.87, 1.32) | 0.50063988940 | 1 |
| **67** | DIACYL-PC C32:0 | 1.06 | (0.88, 1.29) | 0.53283227160 | 1 |
| **68** | DIACYL-PC C42:2 | 0.94 | (0.77, 1.16) | 0.56647765940 | 1 |
| **69** | ACYL-ALKYL-PC C36:3 | 1.05 | (0.86, 1.29) | 0.60192504950 | 1 |
| **70** | DIACYL-PC C34:2 | 1.06 | (0.85, 1.31) | 0.60455689240 | 1 |
| **71** | ACYL-ALKYL-PC C30:2 | 0.94 | (0.75, 1.19) | 0.62234148320 | 1 |
| **72** | ACYL-ALKYL-PC C42:5 | 1.06 | (0.85, 1.32) | 0.63473679760 | 1 |
| **73** | SM C16:0 | 1.05 | (0.86, 1.27) | 0.63665788330 | 1 |
| **74** | DIACYL-PC C38:6 | 0.95 | (0.75, 1.19) | 0.64869962760 | 1 |
| **75** | LYSO-PC C16:0 | 0.95 | (0.76, 1.18) | 0.65345903120 | 1 |
| **76** | ACYL-ALKYL-PC C40:3 | 1.04 | (0.85, 1.28) | 0.67126761130 | 1 |
| **77** | ACYL-ALKYL-PC C40:1 | 0.96 | (0.78, 1.19) | 0.72131021140 | 1 |
| **78** | ACYL-ALKYL-PC C38:3 | 1.03 | (0.85, 1.26) | 0.73292350840 | 1 |
| **79** | ACYL-ALKYL-PC C44:5 | 1.04 | (0.84, 1.28) | 0.73574760340 | 1 |
| **80** | ACYL-ALKYL-PC C34:2 | 0.96 | (0.76, 1.22) | 0.75235043310 | 1 |
| **81** | ACYL-ALKYL-PC C36:0 | 1.03 | (0.83, 1.28) | 0.75387867700 | 1 |
| **82** | ACYL-ALKYL-PC C44:6 | 1.03 | (0.83, 1.29) | 0.75896401250 | 1 |
| **83** | SM C24:1 | 0.97 | (0.79, 1.2) | 0.78319210440 | 1 |
| **84** | Glycine | 0.97 | (0.79, 1.2) | 0.79137116040 | 1 |
| **85** | ACYL-ALKYL-PC C30:0 | 0.97 | (0.77, 1.22) | 0.79602766250 | 1 |
| **86** | SM C16:1 | 0.97 | (0.79, 1.21) | 0.80757844370 | 1 |
| **87** | DIACYL-PC C42:0 | 0.97 | (0.78, 1.22) | 0.81951802920 | 1 |
| **88** | DIACYL-PC C38:5 | 1.02 | (0.83, 1.26) | 0.82802056110 | 1 |
| **89** | DIACYL-PC C32:2 | 0.98 | (0.78, 1.22) | 0.83839610990 | 1 |
| **90** | ACYL-ALKYL-PC C38:5 | 1.02 | (0.83, 1.24) | 0.86870866760 | 1 |
| **91** | DIACYL-PC C42:1 | 0.98 | (0.8, 1.21) | 0.87314725720 | 1 |
| **92** | ACYL-ALKYL-PC C38:6 | 0.98 | (0.79, 1.22) | 0.87780381130 | 1 |
| **93** | SM C18:1 | 1.02 | (0.82, 1.27) | 0.87890466660 | 1 |
| **94** | DIACYL-PC C42:4 | 1.02 | (0.81, 1.27) | 0.88175117930 | 1 |
| **95** | ACYL-ALKYL-PC C44:3 | 0.99 | (0.81, 1.2) | 0.88571036960 | 1 |
| **96** | DIACYL-PC C30:0 | 1.01 | (0.84, 1.22) | 0.91146805550 | 1 |
| **97** | DIACYL-PC C34:3 | 0.99 | (0.8, 1.22) | 0.92000964130 | 1 |
| **98** | SM C20:2 | 0.99 | (0.8, 1.23) | 0.93969905810 | 1 |
| **99** | ACYL-ALKYL-PC C34:1 | 0.99 | (0.81, 1.22) | 0.95264643160 | 1 |
| **100** | Valine | 1.01 | (0.79, 1.28) | 0.95469082090 | 1 |
| **101** | ACYL-ALKYL-PC C40:5 | 1.00 | (0.82, 1.22) | 0.96129383450 | 1 |
| **102** | HYDROXY-SM C22:1 | 1.00 | (0.82, 1.24) | 0.96352448840 | 1 |
| **103** | ACYL-ALKYL-PC C44:4 | 1.00 | (0.81, 1.22) | 0.98375359200 | 1 |
| **104** | DIACYL-PC C40:6 | 1.00 | (0.81, 1.24) | 0.98478384900 | 1 |
| **105** | DIACYL-PC C34:4 | 1.00 | (0.82, 1.22) | 0.99598446240 | 1 |

^a^Metabolites were ranked from lowest to highest raw p-value for the association between metabolites and risk of stroke.

^b^Hazard ratios were derived from Cox regression analysis and refer to standardized log2 transformed metabolite concentrations. The model was adjusted for age, sex, alcohol intake from beverages (non-consumers; women: >0-6 g/d, 6-12 g/d, >12 g/d; men: >0-12 g/d, 12-24 g/d, >24 g/d), smoking (never, former, current ≤20 cigarettes/d, current >20 cigarettes/d), physical activity (Cambridge physical activity index), education (no degree/vocational training; trade/technical school; university degree), prevalent hypertension (yes/no), BMI (kg/m²), waist circumference (cm) and fasting status.

Abbreviations: PC, phosphatidylcholine; SM, sphingomyelin.

**Supplemental Table 3: Selection of serum metabolites associated with risk of myocardial infarction in EPIC-Potsdam (n=2525)**

| **Rank^a^** | **Metabolite** | **Hazard Ratio^b^** | **95% CI** | **Raw p-value** | **FDR p-value** |
| --- | --- | --- | --- | --- | --- |
| **1** | HYDROXY-SM C24:1 | 1.48 | (1.25, 1.76) | 0.0000068729 | 0.0007216564 |
| **2** | HYDROXY-SM C22:1 | 1.58 | (1.26, 2) | 0.0001019915 | 0.0053545539 |
| **3** | ACYL-ALKYL-PC C40:3 | 1.43 | (1.18, 1.73) | 0.0002448504 | 0.0063295814 |
| **4** | ACYL-ALKYL-PC C38:3 | 1.54 | (1.22, 1.94) | 0.0002644205 | 0.0063295814 |
| **5** | HYDROXY-SM C14:1 | 1.48 | (1.19, 1.84) | 0.0003598249 | 0.0063295814 |
| **6** | DIACYL-PC C40:4 | 1.35 | (1.15, 1.6) | 0.0003616904 | 0.0063295814 |
| **7** | SM C24:0 | 1.55 | (1.21, 1.98) | 0.0005307155 | 0.0072675597 |
| **8** | HYDROXY-SM C16:1 | 1.40 | (1.16, 1.7) | 0.0005537188 | 0.0072675597 |
| **9** | ACYL-ALKYL-PC C40:2 | 1.34 | (1.12, 1.6) | 0.0012082151 | 0.0140958423 |
| **10** | DIACYL-PC C40:5 | 1.33 | (1.11, 1.59) | 0.0020081946 | 0.0209822963 |
| **11** | SM C18:0 | 1.43 | (1.14, 1.8) | 0.0021981453 | 0.0209822963 |
| **12** | HYDROXY-SM C22:2 | 1.39 | (1.12, 1.72) | 0.0029907727 | 0.0260738480 |
| **13** | ACYL-ALKYL-PC C36:1 | 1.43 | (1.13, 1.81) | 0.0033862282 | 0.0260738480 |
| **14** | DIACYL-PC C28:1 | 1.35 | (1.1, 1.64) | 0.0034765131 | 0.0260738480 |
| **15** | ACYL-ALKYL-PC C40:4 | 1.31 | (1.09, 1.58) | 0.0037566717 | 0.0262967016 |
| **16** | DIACYL-PC C38:3 | 1.52 | (1.14, 2.03) | 0.0044154804 | 0.0289765904 |
| **17** | ACYL-ALKYL-PC C38:2 | 1.30 | (1.08, 1.57) | 0.0047278780 | 0.0292015994 |
| **18** | ACYL-ALKYL-PC C40:5 | 1.29 | (1.08, 1.55) | 0.0061959306 | 0.0361429286 |
| **19** | ACYL-ALKYL-PC C42:4 | 1.28 | (1.07, 1.54) | 0.0083409938 | 0.0460949660 |
| **20** | DIACYL-PC C42:4 | 1.21 | (1.04, 1.4) | 0.0110677508 | 0.0581056916 |
| **21** | ACYL-ALKYL-PC C34:0 | 1.26 | (1.05, 1.5) | 0.0118634969 | 0.0593174846 |
| **22** | DIACYL-PC C36:1 | 1.35 | (1.06, 1.73) | 0.0155271057 | 0.0741066408 |
| **23** | ACYL-ALKYL-PC C42:5 | 1.24 | (1.04, 1.47) | 0.0179296074 | 0.0775795746 |
| **24** | SM C16:0 | 1.57 | (1.08, 2.28) | 0.0184201444 | 0.0775795746 |
| **25** | DIACYL-PC C30:0 | 1.24 | (1.04, 1.49) | 0.0185132599 | 0.0775795746 |
| **26** | ACYL-ALKYL-PC C38:4 | 1.31 | (1.04, 1.63) | 0.0192101804 | 0.0775795746 |
| **27** | ACYL-ALKYL-PC C36:2 | 1.30 | (1.04, 1.64) | 0.0228519899 | 0.0888688497 |
| **28** | ACYL-ALKYL-PC C42:3 | 1.20 | (1.02, 1.41) | 0.0267779882 | 0.0978119960 |
| **29** | SM C16:1 | 1.36 | (1.04, 1.79) | 0.0270147418 | 0.0978119960 |
| **30** | DIACYL-PC C40:6 | 1.23 | (1.02, 1.48) | 0.0291978782 | 0.0980516258 |
| **31** | DIACYL-PC C38:4 | 1.33 | (1.03, 1.72) | 0.0293216541 | 0.0980516258 |
| **32** | ACYL-ALKYL-PC C38:1 | 1.22 | (1.02, 1.45) | 0.0298824003 | 0.0980516258 |
| **33** | DIACYL-PC C42:5 | 1.15 | (1.01, 1.31) | 0.0329586151 | 0.1048683207 |
| **34** | SM C18:1 | 1.23 | (1.01, 1.48) | 0.0345472880 | 0.1066901540 |
| **35** | ACYL-ALKYL-PC C34:1 | 1.26 | (1.01, 1.56) | 0.0366420233 | 0.1099260700 |
| **36** | SM C20:2 | 1.16 | (1.01, 1.33) | 0.0380949803 | 0.1107249024 |
| **37** | DIACYL-PC C36:2 | 1.54 | (1.02, 2.31) | 0.0397834737 | 0.1107249024 |
| **38** | ACYL-ALKYL-PC C36:3 | 1.24 | (1.01, 1.52) | 0.0404339156 | 0.1107249024 |
| **39** | SM C24:1 | 1.22 | (1.01, 1.48) | 0.0411263923 | 0.1107249024 |
| **40** | ACYL-ALKYL-PC C30:0 | 1.20 | (1.01, 1.43) | 0.0432171873 | 0.1134451167 |
| **41** | ACYL-ALKYL-PC C42:2 | 1.16 | (0.99, 1.36) | 0.0591110285 | 0.1513819022 |
| **42** | DIACYL-PC C32:3 | 1.17 | (0.99, 1.38) | 0.0620553190 | 0.1524605525 |
| **43** | ACYL-ALKYL-PC C40:6 | 1.19 | (0.99, 1.43) | 0.0624362263 | 0.1524605525 |
| **44** | DIACYL-PC C32:0 | 1.20 | (0.99, 1.46) | 0.0645772370 | 0.1541047700 |
| **45** | DIACYL-PC C36:3 | 1.35 | (0.98, 1.86) | 0.0684350495 | 0.1596817823 |
| **46** | ACYL-ALKYL-PC C34:2 | 1.20 | (0.98, 1.46) | 0.0723143492 | 0.1650653622 |
| **47** | ACYL-ALKYL-PC C32:1 | 1.17 | (0.98, 1.39) | 0.0740169615 | 0.1653570417 |
| **48** | ACYL-ALKYL-PC C36:4 | 1.17 | (0.98, 1.38) | 0.0791319427 | 0.1731011246 |
| **49** | ACYL-ALKYL-PC C44:4 | 1.17 | (0.98, 1.4) | 0.0838041272 | 0.1771398121 |
| **50** | DIACYL-PC C34:1 | 1.24 | (0.97, 1.59) | 0.0843522915 | 0.1771398121 |
| **51** | DIACYL-PC C32:2 | 1.16 | (0.97, 1.37) | 0.0990630834 | 0.2039534069 |
| **52** | Arginine | 1.21 | (0.96, 1.54) | 0.1138314669 | 0.2298520004 |
| **53** | DIACYL-PC C34:2 | 1.31 | (0.93, 1.84) | 0.1242456967 | 0.2461471349 |
| **54** | Ornithine | 1.16 | (0.95, 1.42) | 0.1384401545 | 0.2691891893 |
| **55** | Tyrosine | 1.16 | (0.94, 1.43) | 0.1605251933 | 0.3064571872 |
| **56** | Phenylalanine | 1.19 | (0.93, 1.52) | 0.1640731371 | 0.3076371320 |
| **57** | DIACYL-PC C34:3 | 1.12 | (0.95, 1.33) | 0.1693584965 | 0.3119761778 |
| **58** | DIACYL-PC C36:4 | 1.16 | (0.94, 1.42) | 0.1762287972 | 0.3153841298 |
| **59** | ACYL-ALKYL-PC C44:5 | 1.12 | (0.95, 1.31) | 0.1772158443 | 0.3153841298 |
| **60** | DIACYL-PC C38:5 | 1.13 | (0.95, 1.34) | 0.1805224711 | 0.3159143245 |
| **61** | ACYL-ALKYL-PC C38:5 | 1.12 | (0.94, 1.33) | 0.2088887811 | 0.3595626560 |
| **62** | Isoleucine | 1.13 | (0.93, 1.37) | 0.2267103887 | 0.3765282471 |
| **63** | DIACYL-PC C38:6 | 1.11 | (0.94, 1.32) | 0.2269192640 | 0.3765282471 |
| **64** | Valine | 1.15 | (0.91, 1.46) | 0.2295029316 | 0.3765282471 |
| **65** | ACYL-ALKYL-PC C44:6 | 1.11 | (0.94, 1.31) | 0.2346133205 | 0.3789907486 |
| **66** | DIACYL-PC C32:1 | 1.09 | (0.94, 1.26) | 0.2387670452 | 0.3798566628 |
| **67** | LYSO-PC C20:4 | 0.93 | (0.82, 1.06) | 0.2766734678 | 0.4284668691 |
| **68** | ACYL-ALKYL-PC C42:1 | 1.08 | (0.94, 1.26) | 0.2774833057 | 0.4284668691 |
| **69** | Hexose | 1.13 | (0.89, 1.43) | 0.3279384592 | 0.4957284219 |
| **70** | ACYL-ALKYL-PC C38:6 | 1.08 | (0.92, 1.26) | 0.3304856146 | 0.4957284219 |
| **71** | ACYL-ALKYL-PC C36:5 | 1.07 | (0.93, 1.24) | 0.3469787074 | 0.5074674323 |
| **72** | ACYL-ALKYL-PC C32:2 | 1.08 | (0.92, 1.27) | 0.3479776678 | 0.5074674323 |
| **73** | DIACYL-PC C42:0 | 1.09 | (0.91, 1.29) | 0.3585702737 | 0.5157517636 |
| **74** | Histidine | 1.07 | (0.91, 1.25) | 0.4076603817 | 0.5784370281 |
| **75** | ACYL-ALKYL-PC C30:2 | 1.07 | (0.9, 1.27) | 0.4288807550 | 0.5807208069 |
| **76** | LYSO-PC C20:3 | 1.06 | (0.92, 1.22) | 0.4290697985 | 0.5807208069 |
| **77** | LYSO-PC C18:1 | 0.95 | (0.83, 1.08) | 0.4310211039 | 0.5807208069 |
| **78** | LYSO-PC C18:2 | 0.95 | (0.83, 1.09) | 0.4313925994 | 0.5807208069 |
| **79** | DIACYL-PC C34:4 | 1.06 | (0.91, 1.25) | 0.4385030108 | 0.5828204574 |
| **80** | Threonine | 1.07 | (0.9, 1.26) | 0.4523147014 | 0.5872155922 |
| **81** | DIACYL-PC C40:2 | 1.06 | (0.91, 1.22) | 0.4529948854 | 0.5872155922 |
| **82** | LYSO-PC C16:1 | 0.95 | (0.83, 1.09) | 0.4640299280 | 0.5941846639 |
| **83** | Carnithine | 1.06 | (0.9, 1.25) | 0.4743008355 | 0.5962997656 |
| **84** | LYSO-PC C17:0 | 1.06 | (0.9, 1.25) | 0.4770398125 | 0.5962997656 |
| **85** | Tryptophan | 1.06 | (0.9, 1.25) | 0.4911357056 | 0.6066970481 |
| **86** | DIACYL-PC C42:1 | 1.06 | (0.89, 1.27) | 0.5006385674 | 0.6112447625 |
| **87** | ACYL-ALKYL-PC C40:1 | 1.05 | (0.91, 1.21) | 0.5066400050 | 0.6114620750 |
| **88** | DIACYL-PC C38:0 | 1.05 | (0.9, 1.22) | 0.5363862665 | 0.6400063407 |
| **89** | LYSO-PC C18:0 | 1.05 | (0.9, 1.22) | 0.5439910165 | 0.6417871543 |
| **90** | LYSO-PC C28:1 | 1.05 | (0.89, 1.23) | 0.5578511280 | 0.6508263160 |
| **91** | ACYL-ALKYL-PC C36:0 | 1.04 | (0.9, 1.2) | 0.5786473476 | 0.6676700164 |
| **92** | DIACYL-PC C42:2 | 0.96 | (0.82, 1.13) | 0.6245147718 | 0.7114234934 |
| **93** | ACYL-ALKYL-PC C34:3 | 1.04 | (0.89, 1.22) | 0.6301179513 | 0.7114234934 |
| **94** | ACYL-ALKYL-PC C44:3 | 1.04 | (0.89, 1.21) | 0.6515819462 | 0.7278308973 |
| **95** | DIACYL-PC C40:3 | 1.03 | (0.9, 1.18) | 0.6708650588 | 0.7414824334 |
| **96** | DIACYL-PC C36:0 | 0.98 | (0.85, 1.13) | 0.7555474397 | 0.8263800121 |
| **97** | DIACYL-PC C36:5 | 0.98 | (0.84, 1.14) | 0.7672732788 | 0.8305535492 |
| **98** | LYSO-PC C16:0 | 1.02 | (0.89, 1.15) | 0.8052809252 | 0.8628009913 |
| **99** | ACYL-ALKYL-PC C38:0 | 1.02 | (0.88, 1.17) | 0.8216541768 | 0.8714513997 |
| **100** | Proline | 1.01 | (0.88, 1.16) | 0.8635604325 | 0.8891016340 |
| **101** | Glutamine | 1.01 | (0.89, 1.15) | 0.8669166042 | 0.8891016340 |
| **102** | Serine | 1.01 | (0.88, 1.16) | 0.8671112672 | 0.8891016340 |
| **103** | Acetyl carnitine | 0.99 | (0.85, 1.15) | 0.8805468561 | 0.8891016340 |
| **104** | Glycine | 1.01 | (0.87, 1.17) | 0.8888445272 | 0.8891016340 |
| **105** | DIACYL-PC C36:6 | 0.99 | (0.85, 1.16) | 0.8891016340 | 0.8891016340 |

^a^Metabolites were ranked from lowest to highest raw p-value for the association between metabolites and risk of myocardial infarction.

^b^Hazard ratios were derived from Cox regression analysis and refer to standardized log2 transformed metabolite concentrations. The model was adjusted for age, sex, alcohol intake from beverages (non-consumers; women: >0-6 g/d, 6-12 g/d, >12 g/d; men: >0-12 g/d, 12-24 g/d, >24 g/d), smoking (never, former, current ≤20 cigarettes/d, current >20 cigarettes/d), physical activity (cycling and sports in h/week), education (no degree/vocational training; trade/technical school; university degree), prevalent hypertension (yes/no), BMI (kg/m²), waist circumference (cm) and fasting status.

Abbreviations: PC, phosphatidylcholine; SM, sphingomyelin.

**Supplemental Table 4: Selection of serum metabolites associated with risk of myocardial infarction in EPIC-Heidelberg (n=1115)**

| **Rank^a^** | **Metabolite** | **Hazard Ratio^b^** | **95% CI** | **Raw p-value** | **FDR-p-value** |
| --- | --- | --- | --- | --- | --- |
| **1** | SM C24:0 | 1.32 | (1.13, 1.54) | 0.0004797405 | 0.0503727546 |
| **2** | DIACYL-PC C38:3 | 1.34 | (1.13, 1.6) | 0.0007745018 | 0.0813226894 |
| **3** | HYDROXY-SM C22:1 | 1.28 | (1.08, 1.51) | 0.0035269044 | 0.3703249603 |
| **4** | SM C16:0 | 1.25 | (1.06, 1.47) | 0.0069317497 | 0.7278337199 |
| **5** | Serine | 0.77 | (0.64, 0.93) | 0.0077650279 | 0.8153279283 |
| **6** | DIACYL-PC C40:4 | 1.23 | (1.05, 1.44) | 0.0085798498 | 0.9008842317 |
| **7** | DIACYL-PC C36:3 | 1.24 | (1.05, 1.47) | 0.0114607417 | 1 |
| **8** | ACYL-ALKYL-PC C36:4 | 1.23 | (1.04, 1.45) | 0.0164956957 | 1 |
| **9** | ACYL-ALKYL-PC C40:3 | 1.22 | (1.04, 1.44) | 0.0168771873 | 1 |
| **10** | ACYL-ALKYL-PC C36:3 | 1.20 | (1.02, 1.41) | 0.0237423672 | 1 |
| **11** | ACYL-ALKYL-PC C38:3 | 1.20 | (1.02, 1.41) | 0.0297036118 | 1 |
| **12** | ACYL-ALKYL-PC C34:2 | 1.22 | (1.01, 1.46) | 0.0364453828 | 1 |
| **13** | SM C16:1 | 1.19 | (1.01, 1.42) | 0.0428332874 | 1 |
| **14** | ACYL-ALKYL-PC C38:4 | 1.18 | (1.01, 1.39) | 0.0428978280 | 1 |
| **15** | Ornithine | 1.21 | (1, 1.46) | 0.0455794635 | 1 |
| **16** | DIACYL-PC C36:2 | 1.18 | (1, 1.4) | 0.0548268378 | 1 |
| **17** | DIACYL-PC C36:1 | 1.17 | (0.99, 1.38) | 0.0598002344 | 1 |
| **18** | ACYL-ALKYL-PC C38:5 | 1.17 | (0.99, 1.38) | 0.0640921265 | 1 |
| **19** | ACYL-ALKYL-PC C30:2 | 1.17 | (0.99, 1.38) | 0.0650255449 | 1 |
| **20** | DIACYL-PC C40:5 | 1.16 | (0.99, 1.36) | 0.0731754302 | 1 |
| **21** | DIACYL-PC C38:4 | 1.17 | (0.98, 1.38) | 0.0750795825 | 1 |
| **22** | SM C18:0 | 1.16 | (0.98, 1.38) | 0.0770299169 | 1 |
| **23** | SM C24:1 | 1.16 | (0.97, 1.4) | 0.1046198063 | 1 |
| **24** | ACYL-ALKYL-PC C44:4 | 1.15 | (0.96, 1.37) | 0.1234688424 | 1 |
| **25** | LYSO-PC C20:3 | 1.14 | (0.96, 1.37) | 0.1368063752 | 1 |
| **26** | LYSO-PC C17:0 | 0.88 | (0.73, 1.05) | 0.1504925178 | 1 |
| **27** | Tryptophan | 1.14 | (0.95, 1.36) | 0.1553882298 | 1 |
| **28** | DIACYL-PC C42:4 | 1.12 | (0.95, 1.33) | 0.1858249074 | 1 |
| **29** | DIACYL-PC C36:4 | 1.13 | (0.94, 1.35) | 0.1882941826 | 1 |
| **30** | Histidine | 0.89 | (0.75, 1.07) | 0.2117778318 | 1 |
| **31** | Valine | 1.13 | (0.93, 1.37) | 0.2193092396 | 1 |
| **32** | ACYL-ALKYL-PC C40:4 | 1.11 | (0.94, 1.31) | 0.2214299653 | 1 |
| **33** | ACYL-ALKYL-PC C44:6 | 0.89 | (0.74, 1.07) | 0.2264205232 | 1 |
| **34** | DIACYL-PC C34:3 | 1.12 | (0.93, 1.33) | 0.2285882211 | 1 |
| **35** | DIACYL-PC C32:3 | 1.12 | (0.93, 1.34) | 0.2325833098 | 1 |
| **36** | DIACYL-PC C34:2 | 1.11 | (0.93, 1.33) | 0.2452251896 | 1 |
| **37** | SM C20:2 | 1.11 | (0.93, 1.31) | 0.2464009332 | 1 |
| **38** | ACYL-ALKYL-PC C32:1 | 1.10 | (0.93, 1.31) | 0.2666109121 | 1 |
| **39** | SM C18:1 | 1.11 | (0.92, 1.32) | 0.2716685524 | 1 |
| **40** | ACYL-ALKYL-PC C34:1 | 1.10 | (0.93, 1.3) | 0.2772464118 | 1 |
| **41** | ACYL-ALKYL-PC C42:4 | 1.10 | (0.93, 1.3) | 0.2818867279 | 1 |
| **42** | HYDROXY-SM C22:2 | 1.11 | (0.91, 1.35) | 0.3177909135 | 1 |
| **43** | DIACYL-PC C36:6 | 0.92 | (0.78, 1.09) | 0.3246687169 | 1 |
| **44** | ACYL-ALKYL-PC C36:5 | 1.09 | (0.92, 1.3) | 0.3274285389 | 1 |
| **45** | ACYL-ALKYL-PC C40:5 | 1.08 | (0.92, 1.26) | 0.3372642403 | 1 |
| **46** | DIACYL-PC C34:1 | 1.09 | (0.91, 1.29) | 0.3435778209 | 1 |
| **47** | HYDROXY-SM C16:1 | 1.08 | (0.91, 1.29) | 0.3639656977 | 1 |
| **48** | Carnitine | 0.93 | (0.77, 1.11) | 0.4071866775 | 1 |
| **49** | ACYL-ALKYL-PC C38:1 | 1.10 | (0.88, 1.36) | 0.4086687448 | 1 |
| **50** | ACYL-ALKYL-PC C40:2 | 1.08 | (0.9, 1.3) | 0.4186304823 | 1 |
| **51** | ACYL-ALKYL-PC C42:2 | 1.07 | (0.91, 1.24) | 0.4212686866 | 1 |
| **52** | ACYL-ALKYL-PC C38:0 | 0.93 | (0.78, 1.11) | 0.4261219571 | 1 |
| **53** | Glycine | 0.93 | (0.77, 1.12) | 0.4309706424 | 1 |
| **54** | DIACYL-PC C32:0 | 1.07 | (0.9, 1.26) | 0.4415572498 | 1 |
| **55** | ACYL-ALKYL-PC C36:1 | 1.07 | (0.9, 1.26) | 0.4507224851 | 1 |
| **56** | Tyrosine | 1.08 | (0.88, 1.31) | 0.4654642210 | 1 |
| **57** | HYDROXY-SM C14:1 | 1.07 | (0.89, 1.28) | 0.4889227684 | 1 |
| **58** | LYSO-PC C28:1 | 0.94 | (0.78, 1.13) | 0.4999175487 | 1 |
| **59** | HYDROXY-SM C24:1 | 1.06 | (0.89, 1.26) | 0.5012458769 | 1 |
| **60** | ACYL-ALKYL-PC C30:0 | 0.94 | (0.77, 1.14) | 0.5138944065 | 1 |
| **61** | ACYL-ALKYL-PC C44:3 | 1.06 | (0.89, 1.26) | 0.5139771573 | 1 |
| **62** | ACYL-ALKYL-PC C38:6 | 1.06 | (0.89, 1.26) | 0.5169221515 | 1 |
| **63** | LYSO-PC C20:4 | 0.94 | (0.79, 1.13) | 0.5221443144 | 1 |
| **64** | LYSO-PC C18:2 | 0.94 | (0.76, 1.15) | 0.5302256487 | 1 |
| **65** | LYSO-PC C18:1 | 0.95 | (0.79, 1.13) | 0.5440467844 | 1 |
| **66** | ACYL-ALKYL-PC C34:0 | 0.95 | (0.81, 1.12) | 0.5443818664 | 1 |
| **67** | DIACYL-PC C36:5 | 0.95 | (0.81, 1.12) | 0.5544394626 | 1 |
| **68** | Isoleucine | 1.06 | (0.86, 1.31) | 0.5769051837 | 1 |
| **69** | ACYL-ALKYL-PC C40:1 | 0.95 | (0.8, 1.13) | 0.5816434660 | 1 |
| **70** | ACYL-ALKYL-PC C42:1 | 1.05 | (0.89, 1.23) | 0.5902605090 | 1 |
| **71** | DIACYL-PC C32:1 | 1.05 | (0.88, 1.26) | 0.6059323722 | 1 |
| **72** | Proline | 1.05 | (0.86, 1.29) | 0.6195576172 | 1 |
| **73** | DIACYL-PC C38:6 | 0.96 | (0.81, 1.13) | 0.6282660344 | 1 |
| **74** | DIACYL-PC C40:2 | 0.96 | (0.8, 1.14) | 0.6287840123 | 1 |
| **75** | Acetyl carnitine | 1.05 | (0.86, 1.28) | 0.6319853998 | 1 |
| **76** | ACYL-ALKYL-PC C32:2 | 1.04 | (0.87, 1.25) | 0.6336553678 | 1 |
| **77** | ACYL-ALKYL-PC C42:5 | 1.04 | (0.87, 1.25) | 0.6377446936 | 1 |
| **78** | LYSO-PC C16:1 | 0.97 | (0.81, 1.15) | 0.6835056603 | 1 |
| **79** | ACYL-ALKYL-PC C34:3 | 1.03 | (0.87, 1.23) | 0.7066188584 | 1 |
| **80** | DIACYL-PC C38:5 | 1.03 | (0.88, 1.21) | 0.7172869982 | 1 |
| **81** | DIACYL-PC C42:1 | 0.97 | (0.81, 1.16) | 0.7283803688 | 1 |
| **82** | ACYL-ALKYL-PC C44:5 | 0.97 | (0.81, 1.16) | 0.7335265115 | 1 |
| **83** | DIACYL-PC C40:3 | 1.03 | (0.87, 1.22) | 0.7503397249 | 1 |
| **84** | Glutamine | 1.03 | (0.85, 1.24) | 0.7657986796 | 1 |
| **85** | DIACYL-PC C38:0 | 1.03 | (0.86, 1.23) | 0.7660662952 | 1 |
| **86** | DIACYL-PC C42:0 | 0.97 | (0.82, 1.16) | 0.7701156091 | 1 |
| **87** | LYSO-PC C18:0 | 0.98 | (0.82, 1.16) | 0.7994091425 | 1 |
| **88** | ACYL-ALKYL-PC C36:2 | 0.98 | (0.8, 1.19) | 0.8153066452 | 1 |
| **89** | DIACYL-PC C42:5 | 1.02 | (0.87, 1.2) | 0.8218212870 | 1 |
| **90** | ACYL-ALKYL-PC C40:6 | 0.98 | (0.82, 1.18) | 0.8589748280 | 1 |
| **91** | Threonine | 1.02 | (0.84, 1.24) | 0.8616687169 | 1 |
| **92** | DIACYL-PC C36:0 | 0.98 | (0.82, 1.18) | 0.8644463913 | 1 |
| **93** | Phenylalanine | 1.01 | (0.84, 1.22) | 0.8818949378 | 1 |
| **94** | Hexose | 0.98 | (0.77, 1.26) | 0.8961185858 | 1 |
| **95** | ACYL-ALKYL-PC C36:0 | 0.99 | (0.83, 1.18) | 0.9151996389 | 1 |
| **96** | DIACYL-PC C32:2 | 0.99 | (0.83, 1.18) | 0.9213341403 | 1 |
| **97** | DIACYL-PC C30:0 | 0.99 | (0.85, 1.16) | 0.9357714032 | 1 |
| **98** | ACYL-ALKYL-PC C38:2 | 1.00 | (0.84, 1.19) | 0.9593072810 | 1 |
| **99** | DIACYL-PC C34:4 | 1.00 | (0.85, 1.18) | 0.9634736073 | 1 |
| **100** | ACYL-ALKYL-PC C42:3 | 1.00 | (0.84, 1.18) | 0.9659693963 | 1 |
| **101** | DIACYL-PC C28:1 | 1.00 | (0.84, 1.2) | 0.9764134061 | 1 |
| **102** | LYSO-PC C16:0 | 1.00 | (0.83, 1.19) | 0.9792819139 | 1 |
| **103** | Arginine | 1.00 | (0.84, 1.19) | 0.9811185866 | 1 |
| **104** | DIACYL-PC C40:6 | 1.00 | (0.85, 1.17) | 0.9901643548 | 1 |
| **105** | DIACYL-PC C42:2 | 1.00 | (0.85, 1.18) | 0.9939485289 | 1 |

^a^Metabolites were ranked from lowest to highest raw p-value for the association between metabolites and risk of myocardial infarction.

^b^Hazard ratios were derived from Cox regression analysis and refer to standardized log2 transformed metabolite concentrations. The model was adjusted for age, sex, alcohol intake from beverages (non-consumers; women: >0-6 g/d, 6-12 g/d, >12 g/d; men: >0-12 g/d, 12-24 g/d, >24 g/d), smoking (never, former, current ≤20 cigarettes/d, current >20 cigarettes/d), physical activity (Cambridge physical activity index), education (no degree/vocational training; trade/technical school; university degree), prevalent hypertension (yes/no), BMI (kg/m²), waist circumference (cm) and fasting status.

Abbreviations: PC, phosphatidylcholine; SM, sphingomyelin.

**Supplemental Table 5: Hazard ratios across different periods of follow-up time of serum metabolites associated with risk of myocardial infarction**

|  | **EPIC-Potsdam** | | | | **EPIC-Heidelberg** | | | |
| --- | --- | --- | --- | --- | --- | --- | --- | --- |
|  | **Hazard Ratio (95% CI)^a^** | | |  | **Hazard Ratio (95% CI)^a^** | | |  |
|  | **≤3 years FUP**  **N (cases)=65** | **3-6 years FUP**  **N (cases)=65** | **>6years FUP**  **N (cases)=74** | **P for heterogeneity** | **≤3 years FUP**  **N (cases)=66** | **3-6years FUP**  **N (cases)=70** | **>6years FUP**  **N (cases)=92** | **P for heterogeneity** |
|  |  |  |  |  |  |  |  |  |
| **SM C16:0** | 2.00 (1.31; 3.07) | 1.88 (1.22; 2.89) | 1.11 (0.82; 1.50) | 0.0359 | 1.32 (1.02; 1.72) | 1.25 (0.98; 1.59) | 1.13 (0.89;1.43) | 0.6622 |
| **SM C24:0** | 2.14 (1.54; 3.00) | 1.74 (1.24; 2.43) | 1.06 (0.82; 1.35) | 0.0017 | 1.34 (1.04; 1.72) | 1.34 (1.05; 1.70) | 1.20 (0.96; 1.49) | 0.7422 |
| **SM C16:1** | 1.73 (1.20; 2.50) | 1.61 (1.12; 2.32) | 1.02 (0.80; 1.30) | 0.0232 | 1.29 (0.97; 1.72) | 1.20 (0.92; 1.55) | 1.11 (0.87: 1.40) | 0.7090 |
| **Hydroxy-SM C22:1** | 2.09 (1.52; 2.87) | 1.59 (1.15; 2.19) | 1.13 (0.87; 1.49) | 0.0153 | 1.37 (1.02;1.83) | 1.25 (0.97; 1.62) | 1.16 (0.91; 1.49) | 0.7144 |
| **Diacyl-PC C38:3** | 1.56 (1.07; 2.28) | 1.58 (1.10; 2.26) | 1.21 (0.88; 1.65) | 0.4498 | 1.10 (0.82; 1.46) | 1.42 (1.09; 1.85) | 1.33 (1.05; 1.67) | 0.4105 |
| **Diacyl-PC C40:4** | 1.50 (1.15; 1.96) | 1.31 (1.00; 1.71) | 1.17 (0.91; 1.50) | 0.4093 | 1.05 (0.81; 1.36) | 1.21 (0.96; 1.52) | 1.37 (1.10; 1.70) | 0.2951 |
| **Acyl-alkyl-PC C36:3** | 0.98 (0.78; 1.24) | 1.17 (0.81; 1.51) | 1.57 (1.15; 2.13) | 0.0572 | 0.97 (0.74; 1.27) | 1.43 (1.12; 1.83) | 0.99 (0.78;1.26) | 0.0539 |
| **Acyl-alkyl-PC C38:3** | 1.17 (0.88; 1.55) | 1.38 (1.01; 1.87) | 1.67 (1.24; 2.23) | 0.2351 | 1.12 (0.84; 1.48) | 1.30 (1.00; 1.69) | 1.09 (0.86; 1.38) | 0.5940 |
| **Acyl-alkyl-PC C38:4** | 1.03 (0.81; 1.31) | 1.12 (0.82; 1.53) | 1.73 (1.26; 2.36) | 0.0303 | 0.97 (0.74; 1.28) | 1.42 (1.09; 1.86) | 1.07 (0.84; 1.37) | 0.1302 |
| **Acyl-alkyl-PC C40:3** | 1.22 (0.92; 1.62) | 1.29 (0.96; 1.73) | 1.51 (1.58; 1.98) | 0.5302 | 1.27 (0.97; 1.67) | 1.28 (0.99; 1.66) | 1.03 (0.81; 1.30) | 0.3573 |

^a^Hazard ratios were derived from Cox regression analysis and refer to standardized log2 transformed metabolite concentrations stratified by follow-up time (≤3 years; 3-6 years; >6years). The model was adjusted for age, sex, alcohol intake from beverages (non-consumers; women: >0-6 g/d, 6-12 g/d, >12 g/d; men: >0-12 g/d, 12-24 g/d, >24 g/d), smoking (never, former, current ≤20 cigarettes/d, current >20 cigarettes/d), physical activity (Potsdam: cycling and sports in h/week; Heidelberg: Cambridge physical activity index), education (no degree/vocational training; trade/technical school; university degree), prevalent hypertension (yes/no), BMI (kg/m²), waist circumference (cm) and fasting status. Abbreviations: PC, phosphatidylcholine; SM, sphingomyelin.

**Supplemental Table 6. Association between identified metabolites and risk of myocardial infarction with additional adjustment for established CVD biomarkers.**

|  |  | **Hazard ratio per standard deviation^a^** | | | | | | |
| --- | --- | --- | --- | --- | --- | --- | --- | --- |
| **Metabolite** | **Study Population** | **Basic Model** | **+HDL-Cholesterol** | **+ LDL-Cholesterol^b^** | **+Total Cholesterol** | **+Triglycerides** | **+hs-CRP** | **+HDL-Cholesterol**  **+LDL-Cholesterol^c^**  **+Triglycerides**  **+hs-CRP** |
| **Diacyl-PC C38:3** | **Potsdam**  **Heidelberg** | 1.52 (1.14-2.03)  1.34 (1.13-1.60) | 1.60 (1.19-2.15)  1.43 (1.17-1.74) | 1.44 (1.06-1.96)  1.50 (1.17-1.91) | 1.43 (1.03-1.98)  1.54 (1.20-1.98) | 1.51 (1.10-2.08)  1.40 (1.11-1.76) | 1.55 (1.16-2.07)  1.55 (1.27-1.89) | 1.44 (1.03-2.02)  1.40 (1.07-1.82) |
| **Diacyl-PC C40:4** | **Potsdam**  **Heidelberg** | 1.35 (1.15-1.60)  1.23 (1.05-1.44) | 1.38 (1.17-1.64)  1.27 (1.06-1.52) | 1.30 (1.09-1.54)  1.32 (1.06-1.65) | 1.28 (1.07-1.52)  1.34 (1.06-1.68) | 1.32 (1.11-1.57)  1.24 (1.0-1.54) | 1.35 (1.15-1.59)  1.37 (1.15-1.64) | 1.32 (1.10-1.59)  1.22 (1.00-1.57) |
| **Acyl-alkyl-PC C36:3** | **Potsdam**  **Heidelberg** | 1.24 (1.01-1.52)  1.20 (1.02-1.41) | 1.37 (1.09-1.73)  1.32 (1.10-1.59) | 1.18 (0.96-1.44)  1.17 (0.93-1.48) | 1.18 (0.96-1.45)  1.16 (0.91-1.46) | 1.27 (1.03-1.56)  1.22 (1.01-1.48) | 1.28 (1.04-1.56)  1.34 (1.11-1.61) | 1.28 (1.02-1.60)  1.29 (1.01-1.63) |
| **Acyl-alkyl-PC C38:3** | **Potsdam**  **Heidelberg** | 1.54 (1.22-1.94)  1.20 (1.02-1.41) | 1.67 (1.31-2.13)  1.18 (0.98-1.42) | 1.49 (1.17-1.90)  1.16 (0.91-1.46) | 1.49 (1.16-1.92)  1.16 (0.91-1.48) | 1.55 (1.22-1.96)  1.12 (0.90-1.39) | 1.59 (1.26-2.0)  1.24 (1.03-1.50) | 1.56 (1.21-2.02)  1.06 (0.83-1.36) |
| **Acyl-alkyl-PC C38:4** | **Potsdam**  **Heidelberg** | 1.31 (1.04-1.63)  1.18 (1.01-1.39) | 1.41 (1.10-1.81)  1.14 (0.95-1.37) | 1.23 (0.99-1.54)  1.07 (0.85-1.35) | 1.24 (0.99-1.55)  1.08 (0.85-1.37) | 1.33 (1.06-1.66)  1.09 (0.89-1.33) | 1.33 (1.07-1.66)  1.17 (0.98-1.41) | 1.29 (1.02-1.63)  1.03 (0.81-1.31) |
| **Acyl-alkyl-PC C40:3** | **Potsdam Heidelberg** | 1.43 (1.18-1.73)  1.22 (1.04-1.44) | 1.64 (1.32-2.03)  1.21 (1.00-1.46) | 1.39 (1.14-1.70)  1.01 (0.80-1.27) | 1.40 (1.14-1.72)  1.02 (0.80-1.30) | 1.47 (1.21-1.79)  1.06 (0.86-1.30) | 1.49 (1.23-1.81)  1.21 (1.01-1.46) | 1.53 (1.23-1.90)  0.99 (0.78-1.26) |
| **Hydroxy-SM C22:1** | **Potsdam**  **Heidelberg** | 1.58 (1.26-2.00)  1.28 (1.08-1.51) | 1.70 (1.34-2.16)  1.32 (1.09-1.59) | 1.55 (1.18-2.03)  1.08 (0.86-1.36) | 1.57 (1.20-2.04)  1.12 (0.90-1.41) | 1.64 (1.30-2.06)  1.27 (1.05-1.54) | 1.63 (1.30-2.05)  1.30 (1.09-1.56) | 1.52 (1.17-1.99)  1.21 (0.94-1.54) |
| **SM C16:0** | **Potsdam**  **Heidelberg** | 1.57 (1.08-2.28)  1.25 (1.06-1.47) | 1.85 (1.23-2.80)  1.27 (1.07-1.51) | 1.47 (0.94-2.30)  0.92 (0.73-1.16) | 1.51 (0.98-2.34)  0.94 (0.75-1.19) | 1.71 (1.17-2.52)  1.17 (0.97-1.41) | 1.65 (1.14-2.41)  1.22 (1.03-1.45) | 1.55 (0.97-2.48)  1.11 (0.86-1.43) |
| **SM C16:1** | **Potsdam**  **Heidelberg** | 1.36 (1.04-1.79)  1.19 (1.01-1.42) | 1.56 (1.13-2.16)  1.25 (1.04-1.51) | 1.28 (0.96-1.70)  0.92 (0.73-1.16) | 1.30 (0.97-1.74)  0.94 (0.75-1.19) | 1.42 (1.07-1.89)  1.12 (0.92-1.36) | 1.41 (1.07-1.86)  1.16 (0.96-1.39) | 1.35 (0.99-1.84)  1.06 (0.82-1.37) |
| **SM C24:0** | **Potsdam**  **Heidelberg** | 1.55 (1.21-1.98)  1.32 (1.13-1.54) | 1.72 (1.33-2.21)  1.34 (1.12-1.59) | 1.53 (1.14-2.06)  1.02 (0.81-1.30) | 1.56 (1.16-2.09)  1.06 (0.84-1.35) | 1.63 (1.28-2.09)  1.19 (1.0-1.42) | 1.62 (1.27-2.07)  1.35 (1.14-1.60) | 1.52 (1.13-2.03)  1.11 (0.87-1.43) |

^a^Hazard ratios were calculated as a measure of relative risk in continuous models with standardized log2 transformed metabolite concentrations as exposure and incidence of MI as outcome; the basic model was stratified by age and adjusted for sex, alcohol intake, smoking, physical activity, education, fasting status, prevalent hypertension, BMI, waist circumference. Additional models were adjusted for intake of lipid lowering medication and respective biomarkers.

^b^LDL-cholesterol, was estimated using the Friedewald formula .

^c^Results were similar when LDL-cholesterol was replaced by total cholesterol and not different when both were included into the model.

Abbreviation: PC, phosphatidylcholine; SM, Sphingomyelin.

**Supplemental Fig. 1** **Relative contribution of diacyl-phosphatidylcholines C38:3 and C40:4 and acyl-alkyl-phosphatidylcholine C36:3 to predict myocardial infarction in EPIC-Potsdam (a) and EPIC-Heidelberg (b)**

Presented are receivers operating characteristic (ROC) curves comparing different multivariable adjusted models to predict myocardial infarction, using the basic adjustment model as a reference, and adding classical CVD biomarkers (LDL-cholesterol, HDL-cholesterol, triglycerides, hs-CRP) and three metabolites (acyl-alkyl-phosphatidylcholine C36:3 and diacyl-phosphatidylcholines C38:3 and C40:4).

The basic adjustment model included age, sex, alcohol intake, smoking, physical activity, education, fasting status, intake of lipid lowering medication, prevalent hypertension, BMI and waist circumference.

References

1. Boeing H, Korfmann A, Bergmann MM. Recruitment procedures of EPIC-Germany. European Investigation into Cancer and Nutrition. Ann Nutr Metab 1999;43:205-15.

2. Boeing H, Wahrendorf J, Becker N. EPIC-Germany--A source for studies into diet and risk of chronic diseases. European Investigation into Cancer and Nutrition. Ann Nutr Metab 1999;43:195-204.

3. U.S. Department of Health and Human Services, F. a. D. A., Center for Drug Evaluation and Research (CDER), Center for Veterinary Medicine (CVM) (2001). Guidance for Industry. Bioanalytical Method Validation.

4. Floegel A, Drogan D, Wang-Sattler R et al. Reliability of Serum Metabolite Concentrations over a 4-Month Period Using a Targeted Metabolomic Approach. PLoS One 2011;6:e21103.

5. Floegel A, Stefan N, Yu Z et al. Identification of serum metabolites associated with risk of type 2 diabetes using a targeted metabolomic approach. Diabetes 2013;62:639-48.

6. Friedewald WT, Levy RI, Fredrickson DS. Estimation of the concentration of low-density lipoprotein cholesterol in plasma, without use of the preparative ultracentrifuge. Clin Chem 1972;18:499-502.
