# Supplementary figures and images for: Serum metabolites and risk of myocardial infarction and ischemic stroke: a targeted metabolomic approach in two German prospective cohorts
Source: Eur J Epidemiol. 2017 Nov 27;33(1):55–66. doi: 10.1007/s10654-017-0333-0 (PMC5803284; doi:10.1007/s10654-017-0333-0)

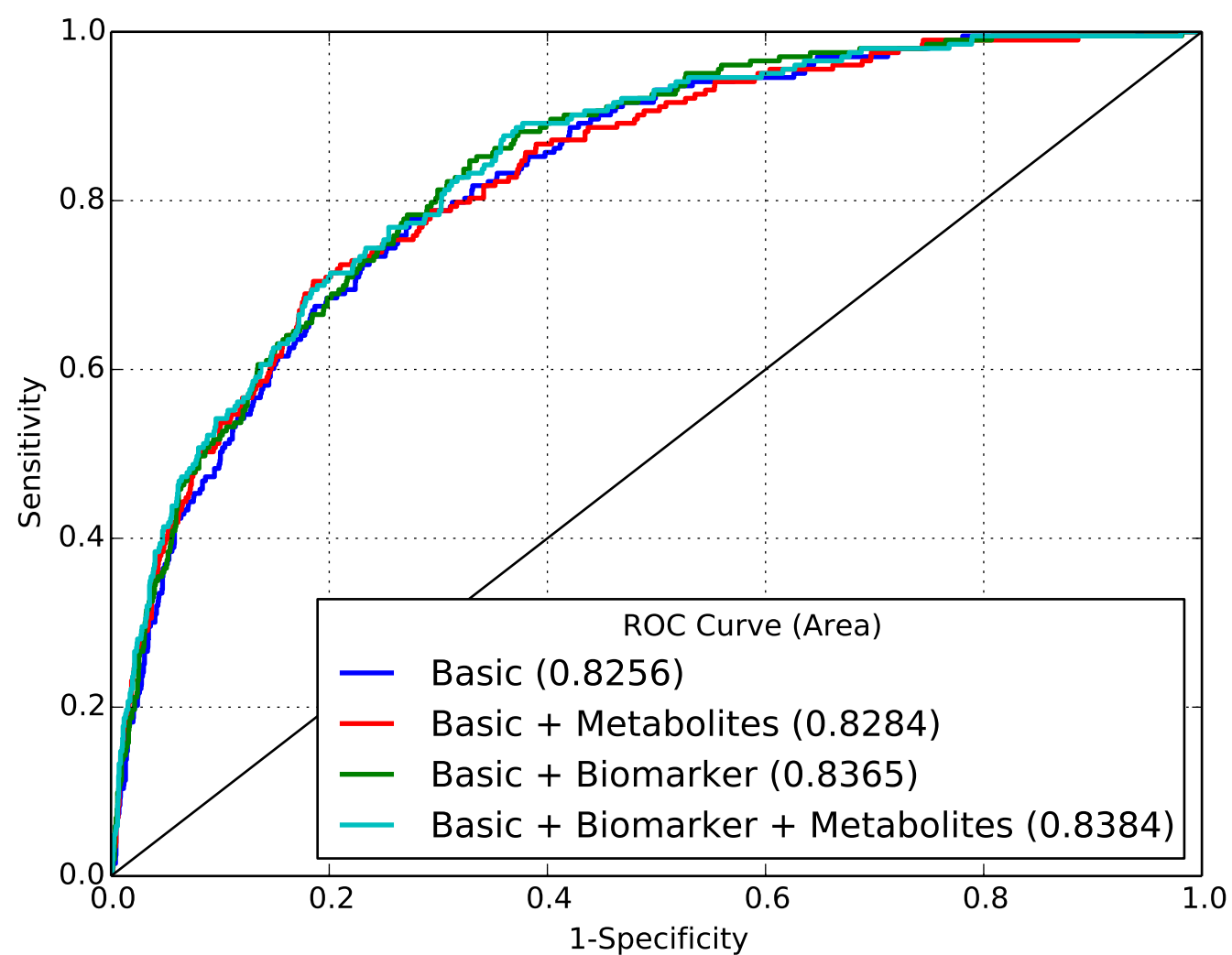

Supplement: Supplementary file 2 — Supplementary material 2 (PDF 22 kb) [file 10654_2017_333_MOESM2_ESM.pdf]

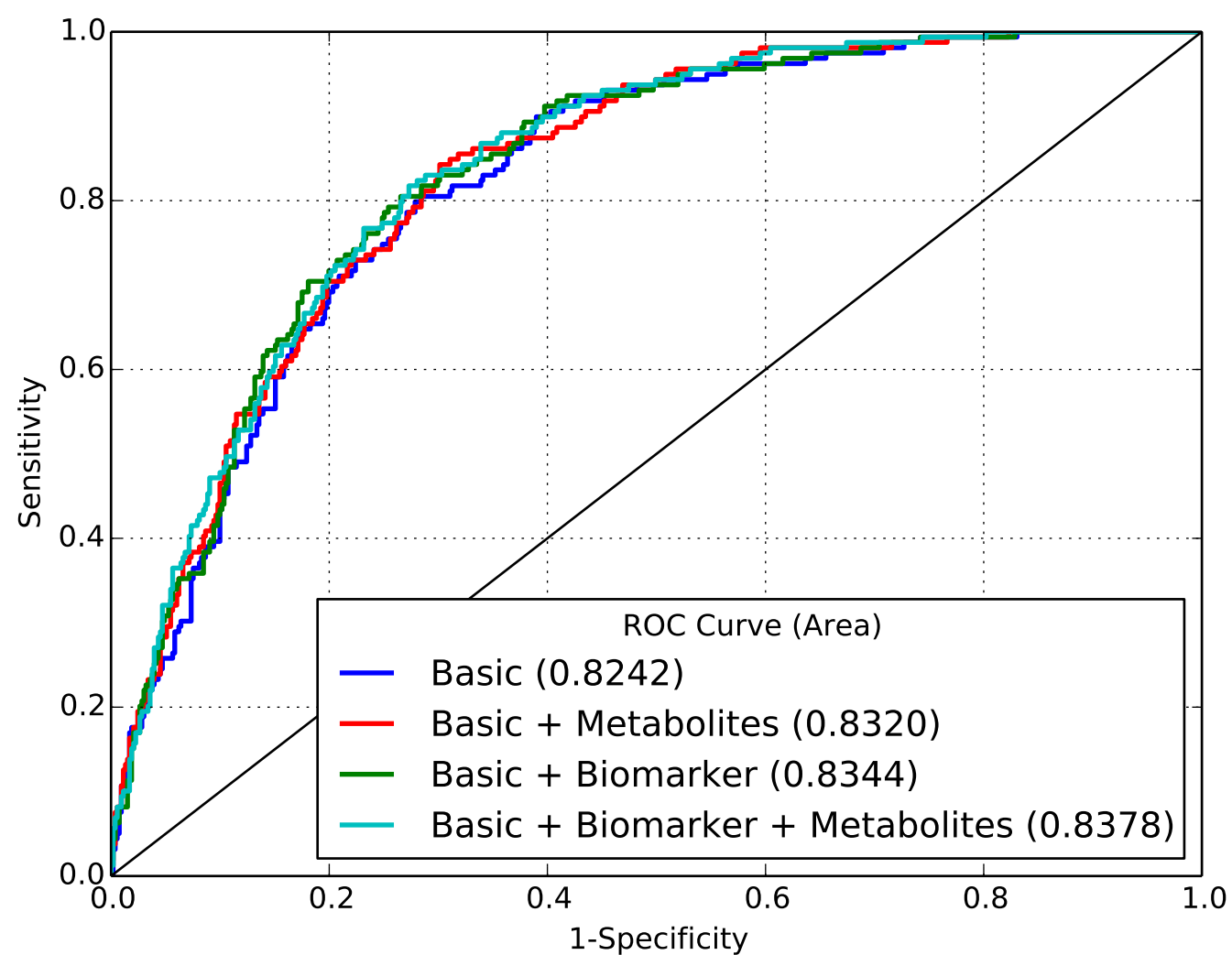

Supplement: Supplementary file 3 — Supplementary material 3 (PDF 19 kb) [file 10654_2017_333_MOESM3_ESM.pdf]
